# FOXO3a (Forkhead Transcription Factor O Subfamily Member 3a) Links Vascular Smooth Muscle Cell Apoptosis, Matrix Breakdown, Atherosclerosis, and Vascular Remodeling Through a Novel Pathway Involving MMP13 (Matrix Metalloproteinase 13)

**DOI:** 10.1161/ATVBAHA.117.310502

**Published:** 2018-01-11

**Authors:** Haixiang Yu, Adam Fellows, Kirsty Foote, Zhaoqing Yang, Nichola Figg, Trevor Littlewood, Martin Bennett

**Affiliations:** From the Division of Cardiovascular Medicine, Addenbrooke's Hospital (H.Y., A.F., K.F., N.F., M.B.) and Department of Biochemistry (T.L.), University of Cambridge, United Kingdom; and Institute of Medical Biology, Peking Union Medical College, Chinese Academy of Medical Sciences, Kunming, Yunnan Province, China (Z.Y.).

**Keywords:** apoptosis, atherosclerosis, downregulation, extracellular matrix, humans

## Abstract

**Objective—:**

Vascular smooth muscle cell (VSMC) apoptosis accelerates atherosclerosis and promotes breakdown of the extracellular matrix, but the mechanistic links between these 2 processes are unknown. The forkhead protein FOXO3a (forkhead transcription factor O subfamily member 3a) is activated in human atherosclerosis and induces a range of proapoptotic and other transcriptional targets. We, therefore, determined the mechanisms and consequences of FOXO3a activation in atherosclerosis and arterial remodeling after injury.

**Approach and Results—:**

Expression of a conditional FOXO3a allele (FOXO3aA3ER) potently induced VSMC apoptosis, expression and activation of MMP13 (matrix metalloproteinase 13), and downregulation of endogenous TIMPs (tissue inhibitors of MMPs). *mmp13* and *mmp2* were direct FOXO3a transcriptional targets in VSMCs. Activation of endogenous FOXO3a also induced MMP13, extracellular matrix degradation, and apoptosis, and MMP13-specific inhibitors and fibronectin reduced FOXO3a-mediated apoptosis. FOXO3a activation in mice with VSMC-restricted FOXO3aA3ER induced MMP13 expression and activity and medial VSMC apoptosis. FOXO3a activation in FOXO3aA3ER/ApoE^−/−^ (apolipoprotein E deficient) mice increased atherosclerosis, increased necrotic core and reduced fibrous cap areas, and induced features of medial degeneration. After carotid artery ligation, FOXO3a activation increased VSMC apoptosis, VSMC proliferation, and neointima formation, all of which were reduced by MMP13 inhibition.

**Conclusions—:**

FOXO3a activation induces VSMC apoptosis and extracellular matrix breakdown, in part, because of transcriptional activation of MMP13. FOXO3a activation promotes atherosclerosis and medial degeneration and increases neointima after injury that is partly dependent on MMP13. FOXO3a-induced MMP activation represents a direct mechanistic link between VSMC apoptosis and matrix breakdown in vascular disease.

## Abstract 2

Supplemental Digital Content is available in the text.

The arterial media comprises vascular smooth muscle cells (VSMCs; ≈30%) and extracellular matrix (ECM, ≈70%), including collagen, elastin, and matrix glycosaminoglycans. The intima in atherosclerosis also comprises both cellular and noncellular components, and advanced plaques show large acellular regions. VSMCs synthesize both filamentous and nonfilamentous ECM components, and chronic low-level VSMC apoptosis promotes medial degeneration characterized by VSMC loss, elastin fragmentation, and ECM degradation.^1^ Medial VSMC apoptosis, elastin fragmentation, and matrix degradation also occur in arterial aneurysms, vascular injury, and vascular remodeling. ECM degradation after VSMC apoptosis may be because of impaired synthesis from fewer VSMCs, increased degradation during VSMC apoptosis, or both processes triggered by the same stimuli. Indeed, macrophages induce VSMC apoptosis and secrete MMP (matrix metalloproteinase) enzymes that degrade ECM in atherosclerosis. However, macrophages are frequently absent in medial degeneration or vascular remodeling, and the mechanisms that might directly link VSMC apoptosis with ECM degradation in vascular disease are unclear.

Apoptosis is regulated via integration of pro and antiapoptotic signals. IGF-1 (insulin-like growth factor 1) is a potent survival factor for VSMCs^2,3^ through activation of the serine/theronine kinase Akt1 (serine threonine kinase of the Akt family). Akt1 protects against VSMC apoptosis and atherogenesis and also promotes fibrous cap formation and reduces necrotic cores.^4,5^ Akt1-induced protection is due in part via phosphorylation of the FOXO (forkhead transcription factor O) subfamily, leading to their association with 14-3-3 proteins and retention in the cytoplasm; survival factor withdrawal leads to FOXO dephosphorylation, nuclear translocation, and activation.^6,7^ The mammalian FOXO subfamily includes 4 members: FOXO1, 3a, 4, and 6.^8,9^ FOXOs are involved in a complicated signaling network, and their transcriptional targets regulate multiple physiological and pathological processes, including cancer, development, mitochondria-dependent and independent oxidative stress, DNA repair, and cell cycle arrest.^10^ However, FOXOs are strictly cell type-specific, and their effects depend on expression levels. For example, FOXO3a protects cells from oxidative stress and DNA damage, but sustained FOXO3a activation leads to apoptosis, and FOXO3a activation induces apoptosis in hematopoietic cells but cell cycle arrest in most other cell types.^11,12^

VSMCs in human atherosclerotic plaques show reduced IGF-1 receptor expression^13^ and phosphorylation of both Akt and FOXO3a, indicating FOXO3a activation.^14^ Massive overexpression of FOXO3a via adenovirus-mediated gene transfer inhibits neointimal hyperplasia by promoting p27 (cyclin dependent kinase inhibitor p27)-mediated cell cycle arrest and apoptosis^11,12^ and prevents VSMC migration through inhibition of CYR61 (cysteine-rich angiogenic inducer 61).^15^ However, the functional consequences of activation of endogenous or low levels of FOXO3a in atherosclerosis, and in particular whether FOXO3a links VSMC apoptosis and ECM degradation, are unknown.

## Materials and Methods

Materials and Methods are available in the online-only Data Supplement.

## Results

### FOXO3a Induces VSMC Apoptosis and MMP13 Expression and Activation

We first generated rat VSMCs expressing N-terminal haemagglutinin-tagged FOXO3aA3ER.^5^ FOXO3a is inhibited by Akt phosphorylation of 3 serine residues, but their mutation to alanine renders FOXO3a insensitive to Akt (FOXO3aA3). FOXO3aA3ER is thus a hydroxytamoxifen-activatable human FOXO3aA3 allele allowing temporal FOXO3a activation. FOXO3aA3ER was inactive without hydroxytamoxifen, but hydroxytamoxifen induced FOXO3a targets such as *bim*, caused FOXO3a translocation to the nucleus within 4 hours, and induced >80% apoptosis within 24 hours, defined by chromatin condensation on DAPI (4’,6-diamidino-2-phenylindole) staining and characteristic appearances on digital videomicroscopy (Figure I in the online-only Data Supplement). To examine downstream signaling after FOXO3aA3ER activation, we examined mRNA expression from FOXO3aA3ER VSMCs and control wild-type rat VSMCs 4 hours after hydroxytamoxifen treatment by microarray^5^ (Table I in the online-only Data Supplement; full data at http://www.bioc.cam.ac.uk/littlewood/foxo3a-microarray/view). Of 27 342 genes, 3467 were upregulated and 4412 genes downregulated 4 hours after hydroxytamoxifen in FOXO3aA3ER VSMCs. In addition to bona fide FOXO3a target genes *(bim*, *gadd45*α, *p27*), other apoptosis and antiapoptosis genes, such as *bcl2*, were upregulated, demonstrating the complexity of FOXO3a regulation of apoptosis. However, several MMPs were also upregulated, and downregulated genes included those associated with ECM attachment and MMP inhibitors. In particular, there was marked induction of *mmp13* (371-fold) and downregulation of *timp3* (27-fold), which would result in markedly increased MMP activity, especially MMP13 (Table I in the online-only Data Supplement).

**Figure 1.**
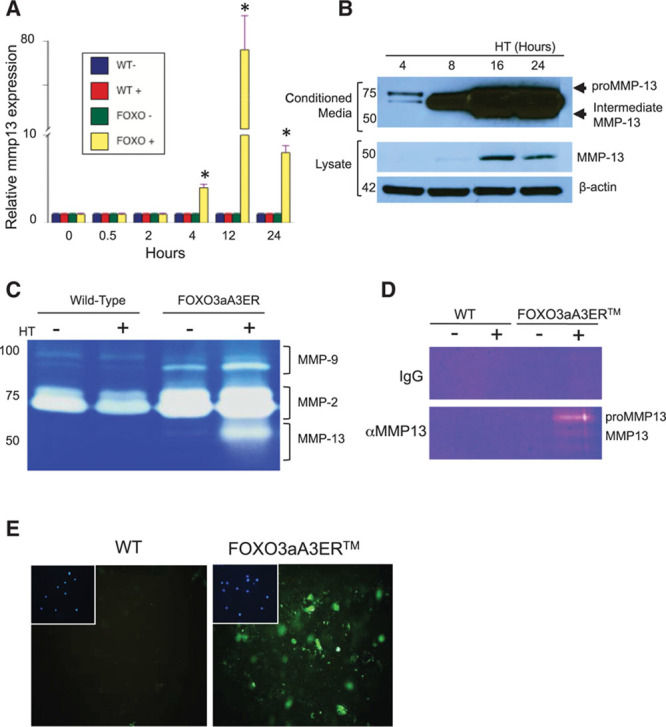
FOXO3a (forkhead transcription factor O subfamily member 3a) activation stimulates MMP (matrix metalloproteinase) expression and activation. **A**, Quantitative polymerase chain reaction for mmp13 mRNA of wild-type (WT) or FOXO3aA3ER vascular smooth muscle cells (VSMCs; FOXO) treated with ethanol carrier control (−) or hydroxytamoxifen (HT; +) from 0 to 24 h. **B**, Western blot of conditioned media (**top**) and cell lysates (**bottom**) of FOXO3aA3ER VSMCs from 4 to 24 h. **C**, Zymogram of MMP activity in conditioned media from WT or FOXO3aA3ER VSMCs after carrier control (−) or HT (+) for 24 h. **D**, Zymogram of MMP13 activity after immunoprecipitation of conditioned medium with IgG control or MMP13 antibody after carrier control (−) or HT (+) for 24 h. **E**, Fluorogenic dye-quenched (DQ) gelatin fluorescence of WT or FOXO3aA3ER VSMCs treated with HT for 16 h. Insets show DAPI (4’,6-diamidino-2-phenylindole) of same field as DQ gelatin. Data are means±SD, n=3. *P<0.001 vs time 0.

To confirm the microarray data, we examined *mmp13* mRNA and protein by quantitative polymerase chain reaction and Western blotting after hydroxytamoxifen treatment ≤24 hours. FOXO3a activation in FOXO3aA3ER VSMCs induced *mmp13* mRNA in a time-dependent pattern (78-fold at 12 hours; Figure 1A), with a lower but significant induction of other FOXO3a targets (*gadd45*α [21-fold], *bim* [18.9-fold], *p27* [11.4-fold], *apaf1* [5.3-fold], *p21* [4.2-fold], and *cyr-61* [3.6-fold]). MMP13 protein expression was increased both in conditioned media and cell lysates, with MMP13 activation demonstrated by protein cleavage from its proform (60 kd) to active intermediate form (Figure 1B). Zymography of the conditioned media confirmed that MMP13 showed the largest change in activity after hydroxytamoxifen (Figure 1C), and immunoprecipitation of the conditioned media with an MMP13 antibody and subsequent zymography showed significantly increased MMP13 activity after hydroxytamoxifen (Figure 1D). In situ zymography showed that hydroxytamoxifen induced degradation of fluorescent-labeled fluorogenic dye-quenched gelatin around FOXO3aA3ER cells (Figure 1E). FOXO3a activation also inhibited expression of TIMP1/2/3 (tissue inhibitors of MMP1/2/3) in cell lysates, with a particularly marked time-dependent reduction in TIMP3 (Figure II in the online-only Data Supplement).

### MMP13 Is a Bona Fide FOXO3a Target in VSMCs

**Figure 2.**
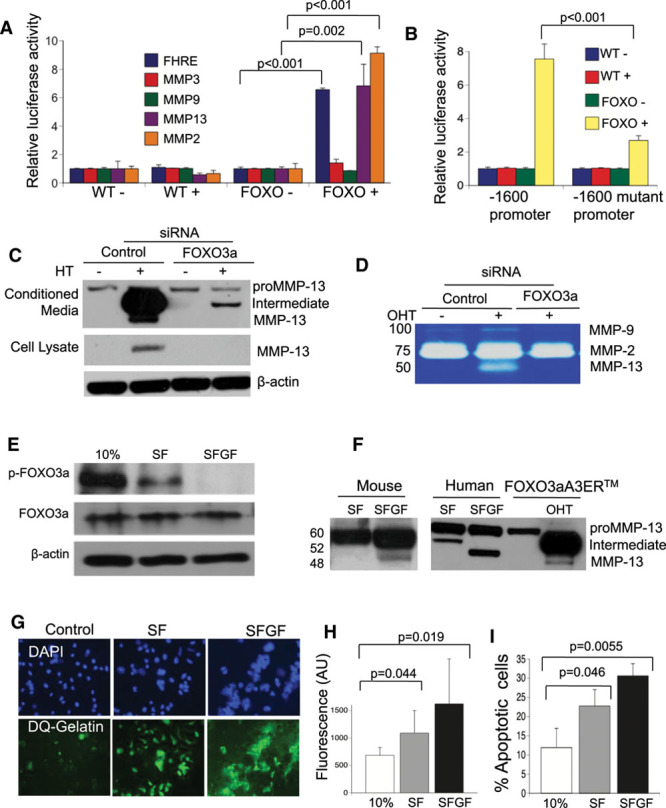
MMP13 (matrix metalloproteinase 13) is a direct target of FOXO3a (forkhead transcription factor O subfamily member 3a). **A**, Dual luciferase assay of wild-type (WT) control or FOXO3aA3ER (FOXO) vascular smooth muscle cells (VSMCs) treated with carrier control (−) or hydroxytamoxifen (HT; +) for 24 h after transfection of plasmids containing a promoter construct containing a forkhead response element (FHRE) or a MMP3, 9, 13, or 2 promoter. **B**, Dual luciferase assay of WT or FOXO3aA3ER (FOXO) VSMCs treated with carrier control (−) or HT (+) after transfection with a −1600 MMP13 promoter fragment or after point mutation of the FOXO3a-binding site. **C** and **D**, Western blot for MMP13 in conditioned media or lysates (**C**), or zymography of conditioned media (**D**) of FOXO3aA3ER VSMCs after transfection with either control siRNA or siRNA to human FOXO3a and 24 h treatment with HT. **E**, Western blot of lysates from human VSMCs incubated in medium containing 10% FCS, 0% FCS (serum free [SF]), or SF and glucose-free medium (SFGF) for 24 h for phosphorylated and unphosphorylated FOXO3a. **F**, Western blot for MMP13 of conditioned media from mouse or human VSMCs in SF or SFGF media compared with FOXO3aER VSMCs±hydroxytamoxifen (OHT) for 24 h. **G**, Fluorescence microscopy for DG-gelatin of human VSMCs in **E**, quantified in **H**. **I**, Percentage of apoptotic cells by flow cytometry of human VSMCs in **E**. Data are means±SD, n=3.

The rapid induction of MMP13 in FOXO3aA3ER VSMCs suggests that MMP13 is a direct FOXO3a transcriptional target. Indeed, *mmp3*, *mmp9*, and *mmp2* promoters show putative FOXO-binding sites, although their activity varies markedly according to cell type.^16,17^ We cotransfected full-length (−1→−1600 promoter region relative to the transcriptional start site) MMP13-, MMP2-, MMP3-, or MMP9-luciferase plasmids with pRenilla-cytomegalovirus into FOXO3aA3ER VSMCs (Table II in the online-only Data Supplement) and a forkhead response element promoter-luc reporter as a positive control. Hydroxytamoxifen induced luciferase activity after transfection with the forkhead response element promoter, full-length MMP13 and MMP2 but not MMP9 and MMP3 constructs (Figure 2A). Point mutation of the FOXO3a DNA-binding motif (Figure III in the online-only Data Supplement) markedly reduced MMP13 promoter activity (Figure 2B). To confirm FOXO3a binding to the MMP13 promoter, FOXO3aA3ER VSMCs were treated with hydroxytamoxifen for 24 hours and chromatin immunoprecipitation performed with either rabbit IgG or a FOXO3a-specific antibody. Hydroxytamoxifen treatment of FOXO3aA3ER VSMCs induced FOXO3a binding to the MMP13 and GADD45α (growth arrest and DNA damage gene) promoters (Figure IV in the online-only Data Supplement). FOXO3a siRNA reduced expression of FOXO3aA3ER and endogenous FOXO3a protein without effects on expression of FOXO1 and FOXO4 (Figure IV in the online-only Data Supplement). FOXO3a siRNA also reduced MMP13 expression in conditioned media and cell lysates (Figure 2C) and MMP13 activity by zymography (Figure 2D), confirming that MMP13 is a target of FOXO3a.

### FOXO3a Activation at Endogenous Levels Induces MMPs and ECM Degradation

Although FOXO3a overexpression studies in rodent cells can elucidate possible FOXO3a targets and functional activity, FOXO3a targets and effects depend on cell type, species, level of expression, and activity. We, therefore, examined the effects of activation of endogenous levels of FOXO3a in human VSMCs by withdrawal of serum±glucose, which reduces Akt-dependent phosphorylation.^13^ Transfer of human VSMCs to serum-free (SF) medium reduced phosphorylated FOXO3a with no change in total FOXO3a; in contrast, complete loss of p-FOXO3a was seen after serum and glucose withdrawal (SFGF), indicating full activation of endogenous FOXO3a (Figure 2E). SFGF induced almost complete nuclear localization of FOXO3a and increased mRNA expression of *bim* (1.5-fold) and *gadd45*α (18-fold), but not *mmp2*, with a 36-fold increase in *mmp13* mRNA (Figure V in the online-only Data Supplement). SFGF also induced FOXO3a binding to the *mmp13* and *bim* promoters (Figure V in the online-only Data Supplement). Compared with SF, SFGF increased MMP13 protein expression and induced MMP13 cleavage in conditioned media of human VSMCs, and mouse VSMCs behaved similarly (Figure 2F). FOXO3a siRNA reduced MMP13 in the conditioned media (Figure V in the online-only Data Supplement). Both SF and SFGF increased gelatin degradation, indicating significant MMP activity (Figure 2G and 2H), and both SF and SFGF induced apoptosis in human VSMCs (Figure 2I).

To ensure that MMP13 induction was not related to lack of glucose in the culture medium, we examined MMP13 in human VSMCs after PI-3 (phosphoinositide 3-kinase) kinase inhibition using LY294002. LY294002 caused complete FOXO3a dephosphorylation in SF medium with no changes in expression of FOXO3a, FOXO1, or FOXO4 (Figure VIA and VIB in the online-only Data Supplement). FOXO3a siRNA was specific for FOXO3a, and LY294002 induced MMP13 expression in the conditioned medium that was reduced by FOXO3a siRNA (Figure VIC and VID in the online-only Data Supplement). Both LY294002-induced MMP13 activity on zymography and apoptosis were reduced by siRNA to FOXO3a, or the MMP13-selective inhibitor WAY170523 (Figure VIE through VIH in the online-only Data Supplement), confirming that activation of endogenous levels of FOXO3a induces MMP13 expression and activity in human VSMCs, which is sufficient to promote apoptosis.

### FOXO3a-Induced Apoptosis Is Mediated, in Part, by MMP13-Induced ECM Degradation and Fibronectin Cleavage

Although FOXO3a induces transcription of many proapoptotic genes, the ECM also provides survival signals, such that degradation of ECM proteins may promote VSMC apoptosis. To determine whether FOXO3a-induced MMP13 degrades ECM survival proteins, we first examined a range of MMP inhibitors on FOXO3a-induced MMMP13 activation and activity, including the broad-spectrum MMP inhibitors GM6001 and Batimastat, the MMP13-selective inhibitors WAY170523 and M13i, and the serine protease inhibitor aprotinin. In particular, WAY170523 has an IC_50_ of 17 nM for MMP13 with >5800-, 56-, and >500-fold selectivity against MMP1, MMP9, and TACE (TNF-α–converting enzyme), respectively.^18^ GM6001, Batimastat and WAY170523 inhibited MMP13 cleavage in conditioned media and activity on zymography, whereas M13i partly reduced MMP13 cleavage but not its activity (Figure VII in the online-only Data Supplement). WAY170523 had minor effects on MMP2 cleavage and activity, but WAY170523 and GM6001 both significantly inhibited fluorogenic dye-quenched gelatin cleavage (Figure VII in the online-only Data Supplement) and apoptosis (Figure VII in the online-only Data Supplement) induced by hydroxytamoxifen treatment of FOXO3aA3ER cells. Our data suggest that WAY170523 is an irreversible inhibitor of MMP13 and that FOXO3a-induced MMP13 provides the primary gelatin-cleaving activity.

The ECM contains proteins that protect VSMCs against apoptosis, including fibronectin and N-cadherin, both of which may be targets of FOXO3a and MMPs,^16,19^ albeit in other cell types and cleaved by different MMPs. Fibronectin is a ligand for surface integrins and has a prominent role in cell adhesion. Hydroxytamoxifen treatment of FOXO3aA3ER VSMCs reduced fibronectin intercellular bridges (Figure VIII in the online-only Data Supplement), and caused fibronectin cleavage, which was inhibited by FOXO3a or MMP13 siRNA, or treatment with WAY170523. Fibronectin also potently protected VSMCs from FOXO3a-induced apoptosis when precoated onto the culture plate. In contrast, FOXO3a activation did not induce N-cadherin cleavage (Figure VIII in the online-only Data Supplement).

### SM22αFOXO3aA3ER Mice

**Figure 3.**
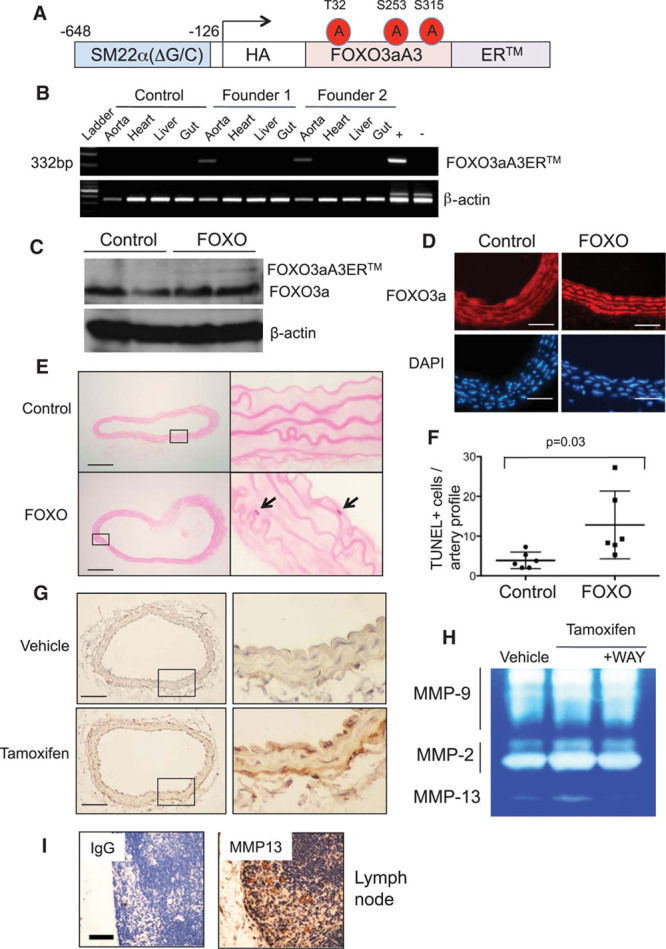
Vascular smooth muscle cell–specific expression of FOXO3aA3ER induces apoptosis in vivo. **A**, Structure of the SM22αFOXO3aA3ER transgene. **B**, Reverse-transcriptase polymerase chain reaction for FOXO3aA3ER in aorta, heart, liver, and gut of 2 SM22αFOXO3aA3ER founder mice or a littermate control. Genomic DNA from SM22αFOXO3aA3ER or littermates were used as positive (+) or negative controls (−). **C** and **D**, Western blot (**C**) or immunohistochemistry (**D**) for FOXO3a (forkhead transcription factor O subfamily member 3a) in littermate control or SM22αFOXO3aA3ER (FOXO) mouse aortas at 7 d after tamoxifen treatment. **E** and **F**, TUNEL (terminal UTP nick-end labeling) of control or SM22αFOXO3aA3ER mouse aortas at 7 d after tamoxifen. **G**, Immunohistochemistry for MMP13 (matrix metalloproteinase 13) in SM22αFOXO3aA3ER mouse aortas after 5 d of vehicle control or tamoxifen. **H**, In situ zymography of SM22αFOXO3aA3ER mouse aortas after 5 d of tamoxifen±WAY170523 (WAY). Scale bars, 25 µm in **D** and 100 µm in **E** and **G**. Data are means±SD, n=6. **I**, Negative and positive controls for MMP13 antibody staining of lymph node. Scale bar, 50 µm. HA indicates haemagglutinin.

To examine the role of FOXO3a in VSMCs in vivo, we generated SM22αFOXO3aA3ER transgenic mice. Haemagglutinin-tagged FOXO3aA3ER was coupled to the minimal SM22α promoter lacking the G/C-rich repressor region (−256 to −249; Figure 3A). This promoter (−447 to +89 relative to the transcriptional start site) directs expression of transgenes only to VSMCs of large- and medium-sized arteries in adult mice, with no expression in venous or visceral SMCs,^20^ whereas G/C-rich region mutation prevents promoter downregulation in atherosclerosis and vessel remodeling.^21^ Two SM22αFOXO3aA3ER founder lines were established, which were viable, fertile, transmitted the transgene normally, and had no overt phenotype without tamoxifen. SM22αFOXO3aA3ER was expressed only in arteries, but not in heart, liver, or gut (Figure 3B), and in cultured VSMCs of transgenic but not littermate control mice. SM22αFOXO3aA3ER activation did not affect expression of other FOXO species but induced *bim*, *gadd45*α, *mmp2*, and *mmp13* and also induced apoptosis (Figure IX in the online-only Data Supplement). Thus, SM22αFOXO3aA3ER is expressed, activated by hydroxytamoxifen, and induces transcription of FOXO3a target genes and apoptosis in VSMCs both in vitro and in vivo.

We treated SM22αFOXO3aA3ER and wild-type littermate control mice with tamoxifen (1 mg immunoprecipitation daily for 3 days) and examined FOXO3a expression, FOXO3a nuclear localization, and apoptosis in aortas at 7 days. FOXO3aA3ER expression was evident in transgenic mouse aortas at low levels compared with endogenous FOXO3a and with no change in expression of endogenous FOXO3a (Figure 3C). Tamoxifen treatment resulted in predominantly nuclear FOXO3a localization in SM22αFOXO3aA3ER mice compared with predominantly cytoplasmic localization in control mice (Figure 3D; quantified in Figure IXD in the online-only Data Supplement). In SM22αFOXO3aA3ER mouse aortas, Tamoxifen induced VSMC apoptosis (Figure 3E and 3F), increased *mmp13* mRNA expression 4-fold, and also induced MMP13 protein expression (Figure 3G) and activity (Figure 3H). MMP13 activity could be inhibited by concomitant treatment with WAY170523, which had no effect on MMP2 or MMP9 activity (Figure 3H).

### FOXO3a Activation Leads to VSMC Apoptosis and Promotes Atherosclerosis

**Figure 4.**
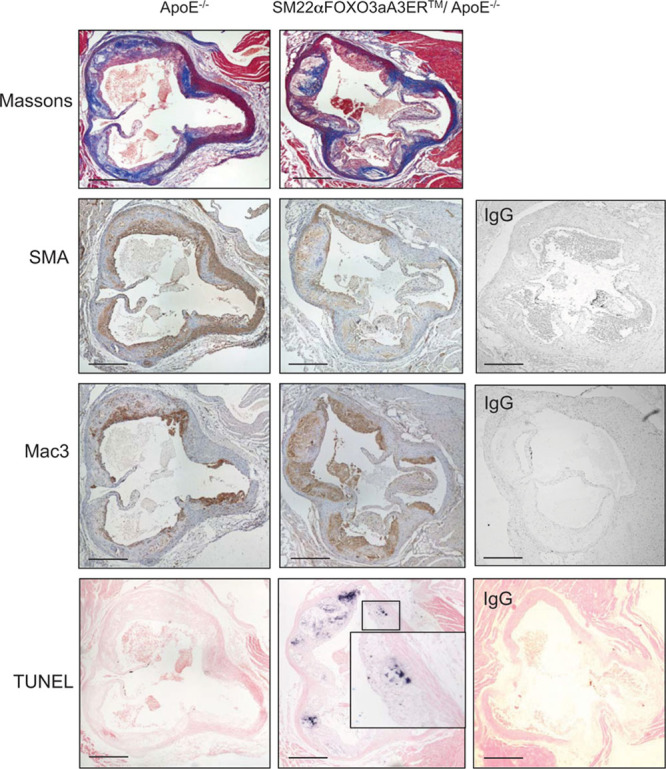
FOXO3a (forkhead transcription factor O subfamily member 3a) activation promotes atherosclerosis, increased necrotic cores, and apoptosis. Histochemistry and immunohistochemistry of aortic root plaques of control ApoE^−/−^ or SM22αFOXO3aA3ER/ApoE^−/−^ mice after 14 wk of fat feeding+tamoxifen. Sections were stained with H+E, Massons, or antibodies to α-SMA (smooth muscle cell α actin), mac3, or underwent TUNEL (terminal UTP nick-end labeling). Scale bar, 500 μm. Insets are high power view of area outlined in the main panel. **Right**, Negative control sections for SMA, mac3, and TUNEL.

**Figure 5.**
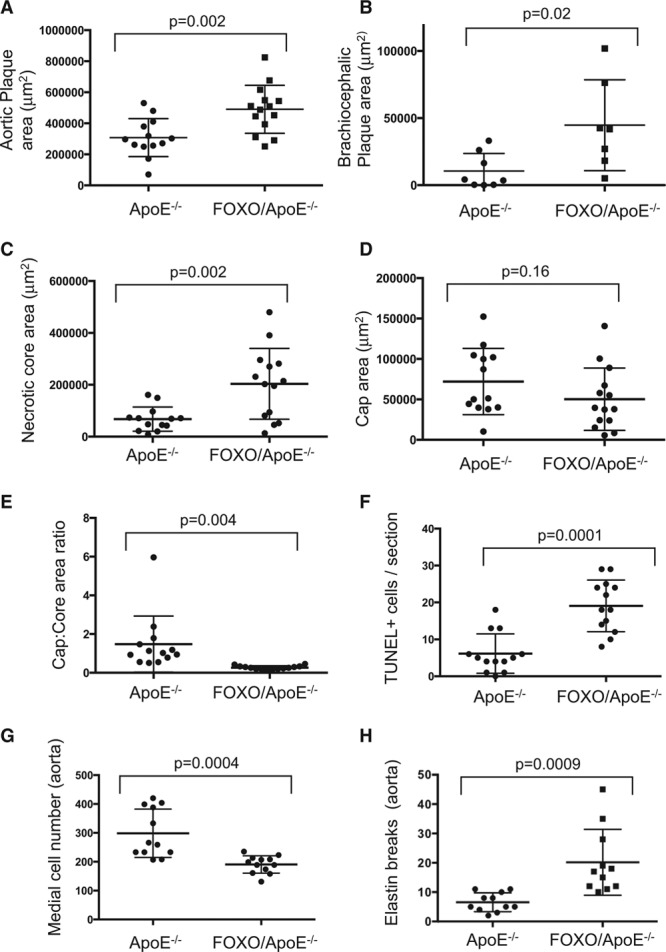
Quantification of size and composition in mouse plaques. **A** and **B**, Plaque size and composition of aortic root (**A**), or brachiocephalic plaques (**B**) of control ApoE^−/−^ or SM22αFOXO3aA3ER/ ApoE^−/−^ mice after 14 wk of fat feeding. n=11 to 15 (aortic), n=8 (brachiocephalic). **C–F**, Plaque composition of aortic root plaques or (**G**) medial cell counts or (**H**) elastin breaks in undiseased aortas of experimental mice. Data are means±SD, n=11 to 14. FOXO indicates forkhead transcription factor O subfamily; and TUNEL, terminal UTP nick-end labeling.

To examine the effect of FOXO3a activation during atherogenesis, SM22αFOXO3aA3ER mice were crossed with ApoE^−/−^ (apolipoprotein E deficient) mice, and homozygous male SM22αFOXO3aA3ER/ApoE^−/−^ or littermate control ApoE^−/−^ mice were fat fed for 14 weeks from 8 to 22 weeks of age. Both groups received hydroxytamoxifen administered triweekly for 14 weeks and bromodeoxyuridine for the last 2 weeks. Serum lipids were measured before and after 7 and 14 weeks of fat feeding. Blood pressure was measured by tail cuff. Atherosclerosis was examined in the aortic roots, brachiocephalic arteries, and descending aorta at 22 weeks (14 weeks of fat feeding), which show different degrees of plaque development. Body weight, serum lipids (Table III in the online-only Data Supplement), heart rate, blood pressure (Table IV in the online-only Data Supplement), and a range of serum inflammatory cytokines (Table V in the online-only Data Supplement) were similar in ApoE^−/−^ and SM22αFOXO3aA3ER/ApoE^−/−^ mice. However, SM22αFOXO3aA3ER/ApoE^−/−^ mice had significantly increased atherosclerosis in all 3 vascular beds (Figures 4, 5A, and 5B; Figure X in the online-only Data Supplement; Table VI in the online-only Data Supplement). SM22αFOXO3aA3ER/ApoE^−/−^ mice had increased necrotic core areas (absolute and core:plaque ratio), reduced relative fibrous cap areas (cap:core and cap:plaque ratios), and more apoptosis (Figure 5C through 5F; Table VI in the online-only Data Supplement). SM22αFOXO3aA3ER/ApoE^−/−^ mice also had reduced aortic media VSMC number, with increased elastin breaks (Figure 5G and 5H; Figure XI in the online-only Data Supplement), but we did not observe aneurysm formation, aortic dissection, or rupture.

### FOXO3a Activation Promotes Apoptosis and Neointima Formation, Mediated, in Part, by MMP13

**Figure 6.**
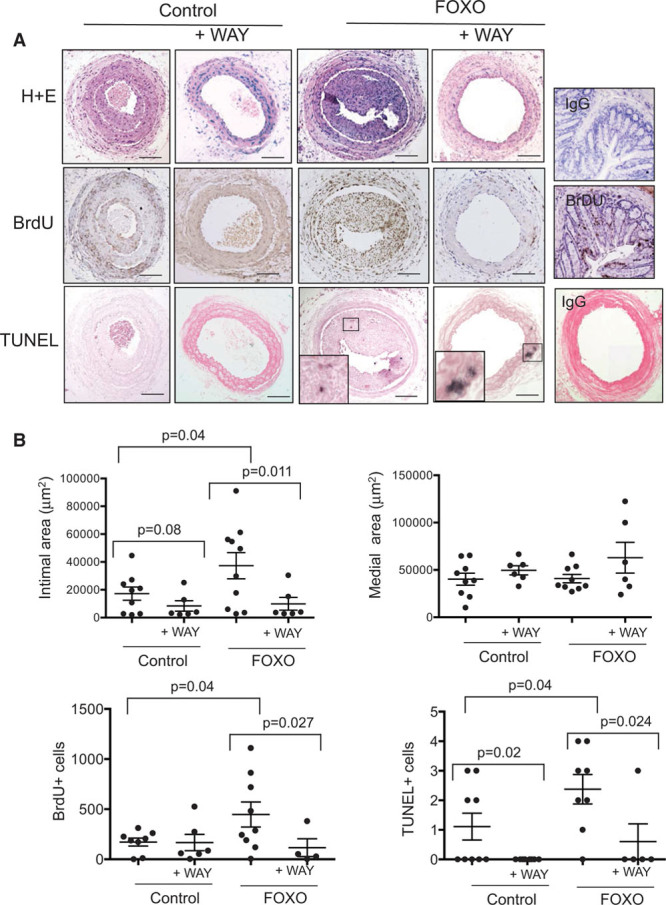
Effects of FOXO3 (forkhead transcription factor O subfamily member 3) activation and MMP13 (matrix metalloproteinase 13) inhibition on arterial remodeling. **A**, Histochemistry and immunohistochemistry of left carotid artery at 28 d post-ligation of SM22αFOXO3aA3ER (FOXO) or littermate control mice, treated with tamoxifen throughout±WAY170523 or vehicle control from 0 to 7 d. Negative and positive control lymph node sections for bromodeoxyuridine (BrdU) or IgG for TUNEL (terminal UTP nick-end labeling) are shown on the (**right**). **B**, Intimal or medial areas, or percentage of cells positive for BrdU or TUNEL in both intima and media in experimental mice as in **A**. Data are means±SEM, n=6 to 11.

Our data indicate that FOXO3a activation induces apoptosis and MMP13 activity in vitro and in vivo that can be inhibited by the MMP13 inhibitor WAY170523 and promotes atherogenesis, increased necrotic core area, reduced relative fibrous cap area, increased apoptosis, and medial degeneration. However, it does not identify whether MMP13 mediates any of the effects of FOXO3a activation in vivo. We, therefore, examined the effect of FOXO3a on remodeling after carotid artery ligation—a well-established model that features medial VSMC apoptosis and MMP activation.^22^ Both final vessel caliber and neointimal size are regulated by VSMC apoptosis in this model,^23^ and the neointima formed is mostly VSMCs derived from the media.^24^ Male SM22αFOXO3aA3ER and control littermate mice aged between 5 and 6 months underwent carotid artery ligation followed by tamoxifen treatment 3× per week to ensure that FOXO3aER is activated throughout the whole time course during remodeling, with or without WAY170523 administered continuously by osmotic minipump from days 0 to 7 after ligation, at a WAY170523 concentration that inhibits MMP13 activity in vivo (Figure 3H). Bromodeoxyuridine was administered throughout. FOXO3a activation increased intimal area and cell count compared with control vessels—an effect that was reduced by WAY170523—with no effect of FOXO3a activation or WAY170523 on lumen area, medial area, or medial cell count (Figure 6; Table VII in the online-only Data Supplement). However, WAY170523 reduced medial cellularity, suggesting that it protects against matrix degradation. FOXO3a activation increased VSMC proliferation, which was also reduced by WAY170523 (Figure 6; Table VII in the online-only Data Supplement). WAY170523 did not affect intimal or medial areas or cell proliferation in the absence of FOXO3a activation in control mice. In contrast, FOXO3a activation in SM22αFOXO3aA3ER mice increased apoptosis compared with control mice, and WAY170523 reduced apoptosis in both control and SM22αFOXO3aA3ER mice (Figure 6; Table VII in the online-only Data Supplement).

## Discussion

FOXO3a activity is increased in VSMCs in human atherosclerotic plaques, and previous studies have demonstrated that Akt1 in VSMCs protects against atherosclerosis^4,5,25,26^; however, the major targets of Akt protection in atherosclerosis are not known. Specifically, FOXO3a has multiple transcriptional targets in a large range of cellular processes, and its effects are cell type specific. Both apoptosis and MMP activity occur in human plaques and often colocalize to the necrotic core, where they have been ascribed to macrophages. However, although VSMCs can generate foam cells in atherosclerosis,^27^ no link between VSMC apoptosis and MMP expression or activity has been shown or the mechanism determined.

There are several important findings from our study. FOXO3a activation markedly induces MMP13 expression and secretion from VSMCs, and MMP13 (but not MMP3 or 9) is a direct FOXO3a transcriptional target in VSMCs. FOXO3a reduces TIMP1-3 expression at both RNA and protein levels, which could further increase MMP activity. FOXO3a activation causes VSMC apoptosis and ECM breakdown, both of which, in part, require MMP13. Importantly, activation of endogenous levels of FOXO3a induces MMP13 expression, secretion, activation, ECM breakdown, and apoptosis, and MMP13 inhibitors reduce FOXO3a-induced apoptosis, confirming that FOXO3a-mediated apoptosis in VSMCs is because of both induction of conventional proapoptotic genes and MMP-induced ECM breakdown of protective proteins, including fibronectin. FOXO3a activation in SM22αFOXO3aA3ER mice induces VSMC apoptosis, increases MMP13 expression and activity, promotes atherosclerosis, increases necrotic core and reduces relative fibrous cap areas, and promotes medial degeneration. Finally, FOXO3a activation in VSMCs increases intimal formation, associated with increased apoptosis and compensatory increased VSMC proliferation; MMP13 inhibition reduces VSMC apoptosis and intimal and medial VSMC proliferation induced by FOXO3a and reduces neointima formation.

The MMPs are 23 secreted or cell surface proteases that hydrolyze ECM components. MMP groups are based on overlapping ECM substrates, for example, collagenase (MMP1, 8, 13), gelatinases (MMP2, 9), and stromelysins (MMP3, 7, 10, 11). MMPs may facilitate VSMC migration and vessel remodeling in early atherosclerosis but degrade ECM in advanced plaques to promote plaque vulnerability.^28^ Expression of some MMPs is increased in atherosclerosis together with reduction in TIMPs in both mice and humans, associated with increased conversion of zymogens to activated proteases.^28^ In particular, atherosclerotic plaques and arterial aneurysms have increased MMP13 activity,^29,30^ and MMP13-null mice have increased fibrillar collagen.^31,32^ Although these findings suggest that MMP13 may be important in atherosclerosis, previous studies have identified macrophages as the likely source,^29^ and MMP13 has not been linked with FOXO3a activation in VSMCs. In contrast, although the absolute increase in *mmp13* mRNA by array or quantitative polymerase chain reaction differed, most likely related to different techniques, we show that FOXO3a activation caused marked release and activity of MMP13 from VSMCs.

The endogenous pathways that protect against VSMC apoptosis in atherosclerosis have not been studied extensively. We find that FOXO3a activation induces apoptosis and ECM degradation through MMP13 in transgenic mice and cultured VSMCs expressing a constitutively active chimeric protein but importantly also with endogenous levels of FOXO3a. Endogenous FOXO3a is activated in human atherosclerosis,^14^ and in culture induces MMP13 induction and secretion, ECM breakdown, and VSMC apoptosis. Endogenous FOXO3a inhibitors are also reduced in atherosclerosis and plaque VSMCs, including Akt^13–15^ and the deacetylase sirtuin 1.^33,34^ Indeed, VSMC-specific SIRT1 deletion promotes atherosclerosis, VSMC apoptosis, and marked medial degeneration, elastin breaks and aneurysm formation in mice.^34^ The combination of reduced FOXO3a inhibitors, increased MMP activation, and reduced TIMP expression induced by FOXO3a would have profound effects on ECM integrity in atherosclerosis in vivo. In addition, as VSMCs synthesize ECM in the vessel wall, FOXO3a-induced VSMC apoptosis might further reduce ECM formation.

We find major effects of FOXO3a in vessel remodeling and neointima formation after carotid artery ligation. This model is characterized by MMP9 activity (days 1–3), VSMC apoptosis (days 2–14), and MMP2 activity (days 14–28), associated with medial recovery, neointimal formation, and arterial remodeling.^22^ Augmenting VSMC apoptosis significantly increases cell proliferation—an example of apoptosis-induced compensatory proliferation, cell migration, and ECM synthesis, resulting in increased neointimal and medial areas.^23^ We find that FOXO3a activation increases neointima formation, intimal and medial proliferation, and VSMC apoptosis, all of which were reduced by MMP13 inhibition, indicating that MMP13 partly regulates the effect of FOXO3a activation on remodeling.

There are several limitations to our study. For example, we used an overexpression system both in vitro and in vivo to examine FOXO3a effects in VSMCs, which may not be subject to the same regulatory controls and, therefore, not truly reflect activation of endogenous FOXO3a. However, the levels of transgene expression in vivo are low, and we show that activation of FOXO3aA3ER and LY294002-induced FOXO3a activation do not result in compensatory changes in expression of other FOXO species. In addition, our studies have focused on the effects of FOXO3a activation in advanced atherosclerosis and have not examined early phases of atherogenesis. However, FOXO3a is not activated until later stages of atherosclerosis,^14^ and VSMC apoptosis is not seen in human atherosclerosis until advanced lesions are present.^35^

The presence of activated FOXO3a in atherosclerosis^14^ and the marked activation and effects of MMP13 might suggest that MMPs in general and MMP13 in particular are attractive therapeutic targets. However, no MMP inhibitor has been approved clinically for atherosclerosis,^36^ which may reflect differing roles of MMPs in plaque development, MMP redundancy with overlapping substrates, and nonspecificity/toxicity of current available inhibitors. Although MMP12 and MMP13 may be more promising targets,^37^ altering the balance of MMPs and TIMPs by inhibiting FOXO3a may be more effective, such as by preventing Akt and SIRT1 downregulation.

In conclusion, we show that FOXO3a activation induces VSMC apoptosis, in part, through regulating MMP13 and MMP inhibitors to promote ECM degradation. Activation of endogenous FOXO3a, as seen in human atherosclerosis, is sufficient to induce apoptosis, MMP activation, and ECM degradation. FOXO3a activation promotes atherosclerosis and medial degeneration, together with increased necrotic cores and reduced relative fibrous cap areas, and FOXO3a and MMP13 regulate arterial remodeling. FOXO3a-induced MMP13 activation and TIMP downregulation provide an important mechanism that directly links VSMC apoptosis and ECM degradation in vascular disease.

## Sources of Funding

This study was funded by British Heart Foundation (BHF) grants (RG/13/14/30314 and PG/11/112/29272), the Oxbridge BHF Centre for Regenerative Medicine (RM/13/3/30159), and the National Institute for Health Research Cambridge Biomedical Research Centre.

## Disclosures

None.

## Supplementary Material

## Notes

Highlights

- The transcriptional factor FOXO3a (forkhead transcription factor O subfamily member 3a) is increased in human atherosclerosis compared with normal vessels, but its role in atherosclerosis and vascular remodeling is unknown.
- FOXO3 activation in vascular smooth muscle cells induces MMP13 (matrix metalloproteinase 13) and reduces MMP inhibitors, causing degradation of the extracellular matrix and survival proteins.
- FOXO3a activation in vivo induces vascular smooth muscle cell apoptosis, promotes atherosclerosis, increases necrotic core, and reduces relative fibrous cap areas.
- FOXO3a activation after arterial ligation promotes neointimal formation, in part, regulated by MMP13.
- The FOXO3a-MMP13 axis directly links vascular smooth muscle cell apoptosis and matrix breakdown in atherosclerosis and vascular remodeling.
