# Supplementary material for: FOXO3a (Forkhead Transcription Factor O Subfamily Member 3a) Links Vascular Smooth Muscle Cell Apoptosis, Matrix Breakdown, Atherosclerosis, and Vascular Remodeling Through a Novel Pathway Involving MMP13 (Matrix Metalloproteinase 13)
Source: Arterioscler Thromb Vasc Biol. 2018 Jan 11;38(3):555–65. doi: 10.1161/ATVBAHA.117.310502 (PMC5828387; doi:10.1161/ATVBAHA.117.310502)
Supplement: Supplementary file 1 [file atv-38-555-s001.pdf]

SUPPLEMENTAL MATERIAL
Detailed Methods
The data, methods used in the analysis, and materials used to conduct the research in this paper will be made available to any researcher for purposes of reproducing the results or replicating the procedure. The microarray data have been made publicly available at (http://www.bioc.cam.ac.uk/littlewood/foxo3a-microarray/view). FOXO3aA3ER^{TM} constructs and SM22α-FOXO3aA3ER^{TM} mice are available on request.

Reagents and antibodies
The following MMP inhibitors and suppliers were used: GM6001 (#364206, Calbiochem) and Batimastat (Millipore), WAY170523 (Cat#2633, Tocris), MMP13i (M13i)(Amgen), Aprotinin (A6279, Sigma). Primary antibodies used for Western blotting were anti-MMP13 (ab39012, abcam), anti-MMP2 (CST4022, Cell Signalling Technologies), anti-FOXO3a/FKHRL1 (sc11351, Santa Cruz); anti-β-Actin (ab8227, ABCAM); anti-P-FOXO1/3a (CST9464, Cell Signalling Technologies), Anti-HA (#11583816001, Roche/Sigma), Anti FOXO1A (ab52857, abcam) and FOXO4 (ab128908, abcam), anti-Bim (ab32158, abcam); Anti-TIMP 1 (AB800, Chemicon), 2 (IM56, EMD Millipore) and 3 (IM43T, EMD Millipore), anti-fibronectin (ab2413, abcam), anti-N-cadherin (ab76057, abcam). Secondary antibodies used were sheep anti-mouse IgG (NA931V) and donkey anti-rabbit IgG, Horseradish Peroxidase-linked (NA934V) from GE Healthcare with 1:2500 dilution.

Generation and characterization of SM22αΔG/C-HA-hFOXO3aA3ER^{TM} (SM22αFOXO3aA3ER^{TM}) transgenic mice
This research has been regulated under the Animals (Scientific Procedures) Act 1986 Amendment Regulations 2012 following ethical review by the University of Cambridge Animal Welfare and Ethical Review Body (AWERB).The transgene SM22αΔG/C-HAhFOXO3aA3ER^{TM}.pA contains a minimal SM22α promoter (-447 to +89) lacking the G/C-rich repressor region (-256 to -249) to drive expression of mutant human FOXO3a (FOXO3aA3) in which three Akt-dependent phosphorylation sites were mutated to alanine (A), with a HA tag and a mutant ligand-binding domain of the mouse estrogen receptor (ER^{TM})[1] linked to the N-and C-terminus of the transgene, respectively. The SM22αΔG/C-HA-hFOXO3aA3ER transgene was assembled in pBluescript, digested with BssHII and the transgene-containing fragment purified from an agarose gel. C57B6/CBA-F1 females were superovulated and paired to F1 stud males. Resulting oocytes were collected and injected with the SM22αΔG/C-HA-hFOXO3aA3ER^{TM} transgene. Transgenic mouse selection was performed after transgene microinjection and two mice that carried the transgene were identified (Founder-1 and -2). The founders were crossed with C57BL/6 mice respectively to generate founder line-derived offspring. Further generations of mice were obtained by crossing the transgene-positive mice with C57BL/6 mice up to 5 times, and subsequently also crossed with C57BL/6 ApoE$^{-/-}$ mice.

Genotyping
DNA extracted from tail or ear biopsies were analysed by PCR with Forward primer 5'-ATGGGAGCTTGGAATGTGAC-3' and Reverse primer 5'-ATAGATCATGGGCGGTTCAG-3' pairs. PCR conditions used were 94˚C for 5 min followed by 35 cycles of 94˚C for 40s, 60˚C for 30s and 72˚C for 40s, with a final extension at 72˚C for 5 min. PCR products were analysed on a 1.5% agarose gel. Genotyping of the ApoE allele was based on the protocol from the Jackson Laboratory (Ma, USA) using a common forward primer (5'-GCC TAG CCG AGG GAG AGC CG-3'), a wild-type specific reverse primer (5'-TGT GAC TTG GGA GCT CTG CAG C) and a mutant reverse primer (5'-x GCC GCC CCG ACT GCA TCT-3').

RT-PCR
Expression of transgene mRNA was determined by RT-PCR. RNA was purified from freshly excised aortas/tissues or cultured cells using TRIzol® RNA Isolation Reagents (15596-026, Life Technologies). The reverse transcription reaction was then undertaken following the manufacturer's Reverse Transcription Protocol (Promega), using oligo(dT)15 primers. The PCR programme used 42°C for 15 mins, 95 °C for 5 mins, and 4 °C for 5 mins.

Quantitative PCR (qPCR)
Pairs of primers were designed and optimized to quantify expression levels of FOXO3aA3ER^{TM} and other genes of interest using quantitative PCR (qPCR). qPCR was performed in triplicate using 2µL cDNA template with SYBR® GreenER™ qPCR SuperMix Universal (11762-100, Life Technologies) and 1µM of both forward and reverse primers in 20µL reaction mixture on a Rotor-Gene 6000 Real-Time Rotary Analyzer (Corbett) as per the manufacturer's instructions, using a standardized cycling programme (94°C for 2 min; 94°C for 10 sec; 40 cycles of 94°C for 15 sec, 61°C for 1 min and 72°C for 1 min; followed by a dissociation stage at 94°C for 15 sec, 60 for 15 sec and 95 for 15 sec). qPCR data were normalized to expression levels of GAPDH in the same samples using Rotor-Gene 6000 analysis software, and are presented as relative expression.

Atherosclerosis studies
Control animals were littermates negative for the SM22αFOXO3aA3ER^{TM} transgene. For atherogenesis studies, SM22αFOXO3aA3ER^{TM} transgenic mice were crossed with ApoE$^{-/-}$ mice (B6.129P2-Apoetm1Unc/J, obtained from Jackson Laboratory). SM22αFOXO3aA3ER^{TM+}/ApoE$^{-/-}$ and control ApoE$^{-/-}$ littermates were fed a high-fat diet (Special Diets Services 829100 -'WESTERN RD') containing 0.15% supplementary cholesterol (giving ~0.2% cholesterol) to promote atherosclerosis from 8-22 weeks of age. Tamoxifen (HT) (T5648, Sigma) was prepared as a suspension in corn oil (C8267, Sigma) and 1mg (100µL) administered by intraperitoneal injection 3 times per week. Mice were euthanized with CO_2. Tissues were removed after perfusion with PBS and post-fixed directly in 10% neutral-buffered formalin. The descending aorta from just distal to the left subclavian artery to the iliac bifurcation was dissected free of connective tissue and fat, cut longitudinally, and mounted en face lumen-side up for Oil Red O staining. Following overnight incubation, tissues were washed and stored in PBS prior to paraffin embedding. Blood samples were taken from overnight-fasted mice and allowed to clot prior to centrifugation (15 min at 8000 x g), serum collected and stored at -80°C. Serum lipids were determined by using a Dimension RXL (Simens) autoanalyser.

Analysis of animal tissues
Sections were stained with hematoxylin and eosin (H+E), Masson's Trichrome or Vehoeff Van Gieson stains. Images were captured with an Axio Imager microscope (Zeiss), and analysed in Image-Pro Insight (Media Cybernetics) to assess atherosclerotic lesion size and composition and areas analysed by planimetry. Fibrous caps were defined as the area rich in VSMCs and proteoglycan overlying the cholesterol-rich, matrix-poor, acellular regions of the necrotic cores. Very small plaques (<90,000µm^2) were not included in the above analysis since it was impossible to demarcate a fibrous cap.

Carotid artery ligation – surgery, tissue preparation and quantification
All animal procedures were performed in accordance with the Animal (Scientific Procedures) Act 1986 and received ethical approval. Male C57BL/6 or SM22αFOXO3aA3ER^{TM} mice were given a pre-operative injection of buprenorphine (0.1 mg/kg; subcutaneously) and anaesthetized with 2.5% inhalable isofluorane. The left common carotid artery was exposed and ligated just below the bifurcation with a 6-0 silk suture. Mice were permitted to recover for 28 days, after which time mice were sacrificed and perfusion-fixed to collect right and left carotid arteries and aorta.

All mice received tamoxifen injections throughout 28 days, and water supplemented with 0.8mg/ml BrdU (B5002, Sigma) and 1% sucrose (S9378, Sigma). Osmotic minipumps (1007D, Alzet) were filled, primed and implanted according to the manufacturer's protocol to deliver WAY170523 (#2633, Tocris) at 7.5mg/kg daily for Days 7-10 after ligation. WAY170523 was initially dissolved in DMSO to a concentration of 100mM before being diluted appropriately using PEG400 such that the final solution contained no more than 50% DMSO.

Immunohistochemistry
Paraffin-embedded tissues were sectioned at 5 µm intervals. Specimens were de-waxed and rehydrated through graded ethanols to water and microwaved in 120mM sodium citrate buffer. Endogenous peroxidase activity was blocked with 3% hydrogen peroxide. Sections were then incubated in 10% bovine serum albumin in PBS for 1 h at RT and then with primary antibodies (MMP13 (ab39012, abcam, 1:250), SMA (M0851, Dako, 1:500), MAC3 (#553322, BD Biosciences, 1:500), BrdU (ab6326, abcam, 1:500) overnight at 4°C. Fluorescent secondary antibodies were applied on the second day. Incorporation of dUTPdigoxigenin (TUNEL assay for apoptosis) was detected with an alkaline phosphatase-conjugated antibody to digoxigenin (Roche) and development with 5-bromo-4-chloro-3indoyl-phosphate/p-nitroblue tetrazolium (Vector). Expression of proteins of interest was determined in three or more random fields, each from at least six different samples.

Zymography of arteries
For protein isolation, whole aortae were excised and removed of peripheral connective tissue before being cut in half into thoracic and abdominal sections. Each portion was placed in Eppendorf tubes then submerged in liquid nitrogen and subsequently stored at -80°C. Immediately after thawing on ice, vessels were transferred to petri dishes containing a small volume of RIPA buffer (#89901, Thermo Fisher Scientific) and chopped finely using a disposable surgical scalpel (#0505, Swann-Morton). Tissues were subsequently homogenized using a TissueLyser LT (2-5 mins at 30-35 rpm, repeated at least 3 times) in RIPA buffer containing two 5mm stainless steel beads (#69989, Qiagen). Samples were then sonicated three times in 10-second bursts at 35% power, and kept on ice in between. Finally, the beads were removed and samples were centrifuged at 12,500rpm for 10 minutes at 4°C to remove debris, then protein concentration assessed by BCA assay.

Gelatin zymography was performed according to the protocol below, except using 20µg protein per lane in pre-cast Novex™ 10% gelatin-containing zymography gels (EC6175BOX, Thermo Fisher Scientific) and gel development was conducted for 5 days rather than overnight. Unless otherwise specified, zymograms were representative of n=3.

Generation of rat FOXO3aA3ER™ VSMCs
VSMCs were derived from male Wistar rats and cultured in DMEM supplemented with 100U/mL penicillin, 100µg/mL streptomycin and 10% FBS. Cells were infected with an ecotropic retrovirus expressing FOXO3aA3ER™ in the presence of 8µg/mL polybrene and selected with the appropriate antibiotic. Expression of the exogenous protein was verified by Western blotting.

Microarrays
mRNA expression from FOXO3aA3ER™ VSMCs was compared with control rat VSMCs 4hr after HT treatment by microarrays as previously described[2].

Isolation and culture of VSMCs from Aorta
VSMCs were isolated from mouse or normal human aorta by digesting with 1mg/ mL of collagenase (C0130, SIGMA) and 0.5mg/mL of elastase (LS006365, Worthington) in serum-free Dulbecco's Modified Eagle Medium (DMEM) (D5671, SIGMA), supplemented with 2mM of L-glutamine (G7513, SIGMA), 100 units/ mL of penicillin and 100µg streptomycin (P0781, SIGMA). Cells were further passaged and maintained in complete DMEM with 10% FCS.

Luciferase reporter assay
Cells were seeded at 5×10^{5} cells per well in 6-well plates for 24 hours and allowed to attach overnight. The relevant experimental plasmids were cotransfected with pRenilla-CMV (Promega, UK) reference plasmid using X-tremeGENE 9 DNA transfection reagent (Roche, Switzerland) following the manufacturer's protocol. Cells were incubated at 37°C for 24 hours and then stimulated with or without HT (1µM in ethanol) for a further 24 hours. Cell lysates were prepared and simultaneously assayed for firefly and renilla luciferase activity using the Dual luciferase Assay System (Promega, UK) on a Turner luminometer. Firefly luciferase activity was normalized for the Renilla luciferase activity. Values are expressed as fold activation relative to unstimulated cells

Small interfering RNA (siRNA) for gene silencing
Expression of FOXO3a was inhibited using SMARTpool siRNA (Dharmacon) containing four individual siRNAs per gene, and a 'scrambled' control siRNA as a negative control. Cells were transfected with siRNA using the Dharmafect transfection reagent 1 (T-2001-02, Dharmacon) as per the manufacturer's instructions. Cell lysates were prepared 48 h post-transfection in RIPA buffer and protein expression levels determined by Western blotting.

Western blotting
Equal amounts of protein were resolved on acrylamide:bis-acrylamide gels and electroblotted onto PVDF membrane (Immobilon-P, Millipore). Membranes were blocked in 5% Marvel dried skimmed milk in TBS-T (Tris-buffered saline supplemented with 0.1% Tween 20) for 1h on a rocking platform at room temperature and incubated with primary for 2h prior to incubation with the appropriate horseradish peroxidase (HRP)-conjugated secondary antibody. Antibody binding was determined by enhanced chemiluminescence (GE Healthcare). Unless otherwise specified, western blots were representative of n=3.

Determination of apoptosis in vitro
Apoptosis was determined either by time-lapse microscopy or Annexin V staining. For time-lapse, VSMCs were seeded into 12-well plates in standard media supplemented with 40mM N-2-Hydroxyethylpiperazine-N-2-Ethanesulfonic Acid (HEPES, Sigma) for 1h. The plates were sealed and mounted on an Olympus IX-70 microscope fitted with automated shutters and motorized stage all controlled by Openlab software (Perkin Elmer). Images were captured at 4h intervals and 100 cells per field (3 fields) analysed for cell division and apoptosis. Apoptosis was verified by morphological changes such as cell shrinkage and membrane blebbing. Results are expressed as cumulative cell divisions or cumulative cell deaths. Apoptosis was also determined using the Annexin V apoptosis detection kit (BioVision) as per the manufacturer's instructions and the number of apoptotic and necrotic cells quantified by Flow Cytometry (Accuri C6).

Chromatin immunoprecipitation (ChIP)
Cells were seeded into T150 culture flasks and allowed to grow in complete DMEM to around 90% confluency. Cells were then treated with 1µM HT for 24h and ChIP performed with either rabbit IgG or rabbit anti FOXO3a (FKHRL1 (H-144**)**, sc-11351, Santa Cruz Technology, UK) using EZChIP kit (17-371, Merck Millipore, UK) according to the manufacturer's instructions. Briefly, cells were cross-linked with formaldehyde (Sigma F8775) to a final concentration of 1% for 10 mins at RT, and then neutralized with glycine (1.25 M) for 5 min at RT. Cells were washed twice with ice-cold PBS, resuspended in SDS lysis buffer containing Complete Protease Inhibitor Cocktail (Roche) and sonicated for 15 mins using a Bioruptor UCD200 system. Sheared samples were centrifuged for 10 mins at 14,000 rpm at 4°C. Supernatants were immunoprecipitated with antibodies against rabbit anti-FOXO3a (3µl per 300µg chromatin) or rabbit IgG control at 4°C overnight. Protein A/G magnetic beads (50:50 slurry, 60µL/sample) were added and incubated at 4°C for 1 hour. The beads were then washed with 1x low salt, 1x high salt, 1x LiCl buffer and 2x TE buffer, and immunoprecipitated DNA complexes eluted using 200µL elution buffer (50mM sodium bicarbonate, 1% SDS). Reverse cross-linking was performed by adding NaCl (5M) and incubating at 65°C for 4 hours. Complexes were treated with RNase and protease K for 30 mins at 37°C and 2 hours at 45°C sequentially, and immunoprecipitated DNA-protein complexes purified with spin columns (Upstate, USA) and used for qPCR amplification. Fold enrichment of each promoter region was expressed as a ratio of PCR signal of samples to that of input. Regions of interest were amplified from the immunoprecipitated DNA by qPCR using SYBR GreenER qPCR SuperMix Universal. The primers for the rat mmp13 −204/−34 were: forward, 5′-CAGATGCGTTTTGATATGCC-3′; and reverse, 5′AATAGTGATGAGTCACCACTT-3′ and primers for rat gadd45α -332/+23: forward 5'-AGAAACAATTGGCAGGCTGT-3'; and reverse 5'-GGCTTTTGGAGTGGTTCAAG -3'.). The ChIP primers for mouse mmp13 and bim were forward, 5′-CTGCTGCTTCTCCCCACTAT-3′, reverse, 5′-TGAGGCGAAGGTAAACATGC-3′; forward, 5′-GGGCGGGTACATTCTGAGT-3′, reverse, 5′-CAGGCTGCGACAGGTAGTG-3′. Fold enrichment of each promoter region was expressed as a ratio of PCR signal of samples to that of input.

Immunocytochemistry (ICC)

Cells were seeded at 1.5 x 10^4 cells per well in 4-well culture chamber plates (BD-UK) and grown in complete DMEM. After 20 hours cells were stimulated with HT (2 µM in ethanol) or medium control and incubated for 4 or 16 hours. Briefly, cells were fixed with -20°C methanol for 10 mins at RT, washed twice with warm PBS (1 ml) for 5 mins at RT, and permeabilized with PBS containing 0.25% Triton-X100 (9002-93-1, Sigma-Aldrich) for 10 mins at RT. After further washing with PBS, 3x 5 mins, cells were incubated with normal goat serum (10% in 0.1% Tween-20 in PBS (PBST))(Dako) for 1 hour at RT. The primary antibody, either anti-ER (0.5% (v/v)) in 1% BSA/PBST or anti-MMP13 (0.5% (v/v)) in 1% BSA/PBST, was incubated for 16 hours at 4°C and 1 hour at RT respectively. Cells were then washed with PBST at RT, 3x 5 mins, before adding the secondary goat anti-Rabbit ALEXA 594 antibody (0.1% (v/v)) (A11012, Invitrogen) in 1% BSA/PBST and incubated for 1 hour at RT. Cells were then extensively washed with PBST. Finally 4',6-diamidino-2-phenylindole (DAPI) (100ng/ml in PBS) (D-1306, Molecular Probes) was added and incubated for 30 mins at RT. The cells were washed with PBS, mounted using mounting medium without DAPI (5µl) (H-1000, Vector Laboratories, Burlingame). Viewing and images were taken using an Olympus U-RFLT fluorescence microscope, and CC12 Soft Imaging System, using the software Cell^D.

Site-directed mutagenesis

Site-directed mutagenesis was carried out on the -1600 -+1 pMMP13-luc plasmid, forward primer 5'-AAAAAGTCGCCACGTAAGCATGCTTACCTTCAAGTGACTAGGAAG-3' and reverse primer 5'-CTTCCTAGTCACTTGAAGGTAAGCATGCTTACGTGGCGACTTTTT-3', using pfu polymerase. The DNA was then transformed into Top10 E. Coli cells following the Heat shock Protocol and plated out on LB agar containing Ampicillin. Single colonies were picked, grown overnight, and plasmids prepared using a QIAprep Spin Mini-prep kit (Qiagen, UK). The mutated promoter sequence was confirmed by sequencing analysis before use.

Immunoprecipitation

Supernatants of wild type control (WT) and FOXO3aER cells, stimulated with ethanol carrier or HT (2µM in ethanol) for 24 hours, were collected and concentrated using an Ultracel 10k concentrator, spun at 5000 rpm for 7 mins to a volume of about 400µl. Each sample was split equally into two. Rabbit IgG antibody (0.25% (v/v)) or anti-MMP13 antibody (1% (v/v)) were added and incubated at 4°C with gentle shaking overnight. Dynabeads® Proteins A/G for immunoprecipitation (1:1 ProteinA:ProteinG) (Invitrogen) were washed in RIPA buffer (10mM Tris-HCL pH7.5; 1mM EDTA; 1% Triton-X100, 0.1% SDS, 0.1% Sodium Deoxycholate, 150mM NaCl, Complete Protease Inhibitor Cocktail), added to each sample (40µl) and incubated at 4°C with shaking for 1 hour. The beads were then washed with 3x wash buffer (50mM Tris pH 8.1, 10mM EDTA pH 8.0), and MMP13 protein eluted in non-denaturing sample buffer (50µl).

Gelatin Zymography

A 10% SDS-PAGE running gel was prepared containing gelatin (1mg/ml)(9000-70-8, Sigma-Aldrich). Each sample was loaded (20µl) and the gel was run at 60-100 V for 2-3 hours. The gel was then incubated in 2.5% Triton-X100 with shaking for 30 mins at RT, washed with 1x zymography developing buffer (Invitrogen) with shaking for 30 mins, then left overnight in fresh zymography developing buffer at 37°C. The gel was stained with Coomassie Blue R250 (0.5%) for 60 mins, then destained with Coomassie R-250 destaining solution (Methanol:Acetic Acid:Water (50:10:4)).

DQ Gelatin assay

Cells were plated onto 4-well chamber slides and after treatment were washed in PBS before applying 1mg/ml DQ-gelatin (Thermo Fisher Scientific) for 3 hours at 37°C. Cells were then washed again with PBS and mounted on coverslips. Nuclei were determined with DAPI staining whilst DQ-gelatin degradation was visualized by detection of green fluorescence at 488nm and quantified using ImageJ software.

Statistical analysis

Data were tested for a normal distribution by examination of the histogram and normal

probability plot, and by using the Shapiro-Wilk test. Student's t test was used for pair-wise comparison for data following a normal distribution, and Mann Whitney U test for non-parametric data. ANOVA with Bonferroni's correction was used to compare means of multiple groups. Values are presented as means ± SD unless otherwise specified. $p<0.05$ was considered significant.

References

1. Littlewood T, Hancock D, Danielian P, et al. A modified estrogen-receptor ligand-binding domain as an improved switch for the regulation of heterologous proteins. *Nucl Acid Res*. 1995;23:1686-1690.
2. Tucka J, Yu H, Gray K, et al. Akt1 regulates vascular smooth muscle cell apoptosis through FoxO3a and Apaf1 and protects against arterial remodeling and atherosclerosis. *Arterioscler Thromb Vasc Biol*. 2014;34:2421-8.
