# Supplementary material for: FOXO3a (Forkhead Transcription Factor O Subfamily Member 3a) Links Vascular Smooth Muscle Cell Apoptosis, Matrix Breakdown, Atherosclerosis, and Vascular Remodeling Through a Novel Pathway Involving MMP13 (Matrix Metalloproteinase 13)
Source: Arterioscler Thromb Vasc Biol. 2018 Jan 11;38(3):555–65. doi: 10.1161/ATVBAHA.117.310502 (PMC5828387; doi:10.1161/ATVBAHA.117.310502)
Supplement: Supplementary file 2 [file atv-38-555-s002.pdf]

Supplemental Figures and Tables

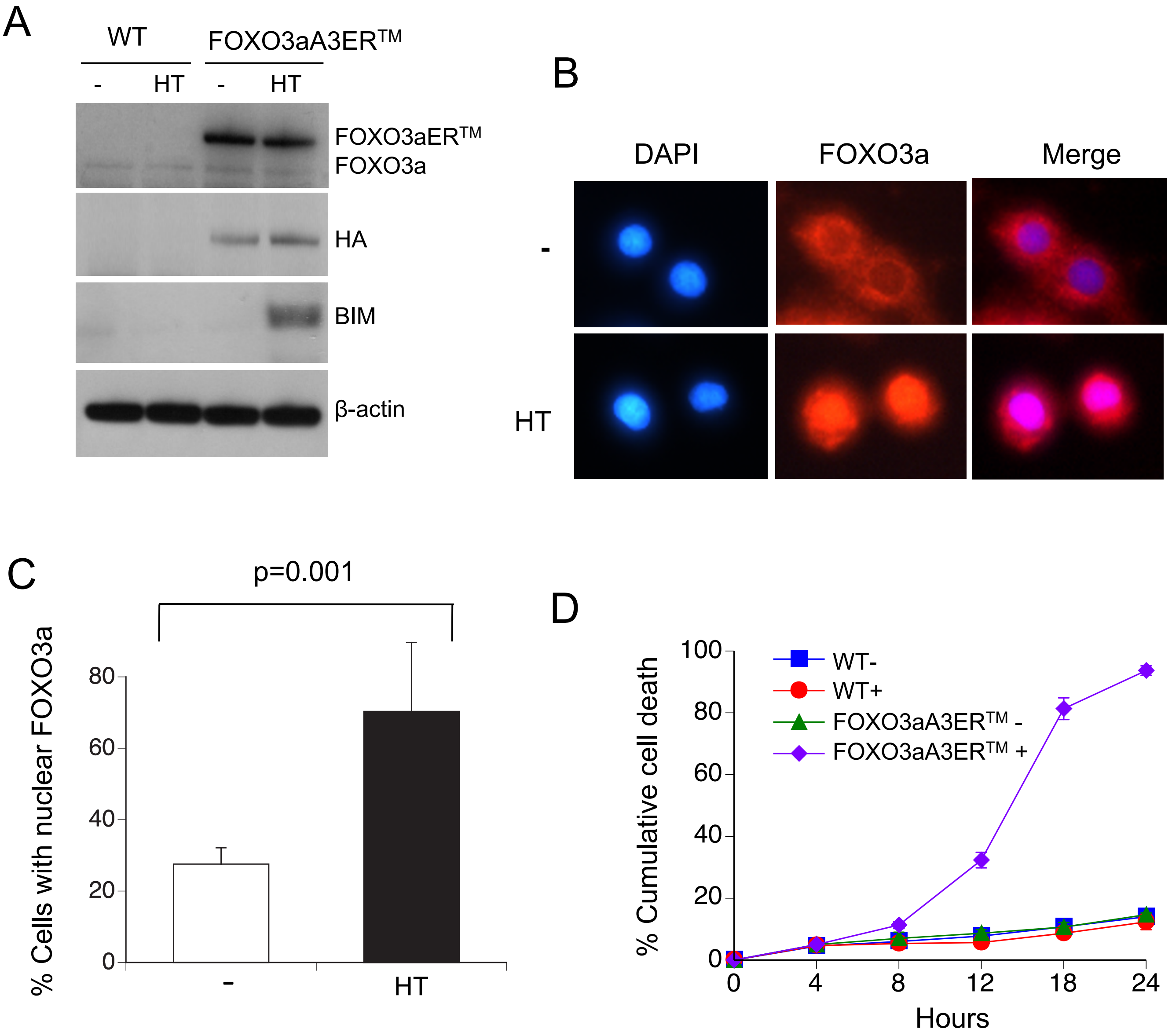

Supplemental Figure I

HT activates FOXO3a and leads to VSMC apoptosis

(A) Western blot of wild type control (WT) or FOXO3aA3ER^{TM} VSMCs for FOXO3a, HA or the FOXO3a transcriptional target BIM after administration of hydroxytamoxifen (HT) vs. the ethanol carrier control (-). **(B)** Immunocytochemistry for FOXO3a (red) or DAPI (blue) with overlay (pink) in FOXO3aA3ER^{TM} VSMCs treated with 1 μM HT or carrier control (-) for 4h. **(C)** Quantification of % cells expressing nuclear FOXO3a in **(B)**. **(D)** Digital videomicroscopic analysis of % cumulative cell death in WT or FOXO3aA3ER^{TM} VSMCs after 1 μM HT (+) or carrier control (-) up to 24h. n=3, data are Means±SD.

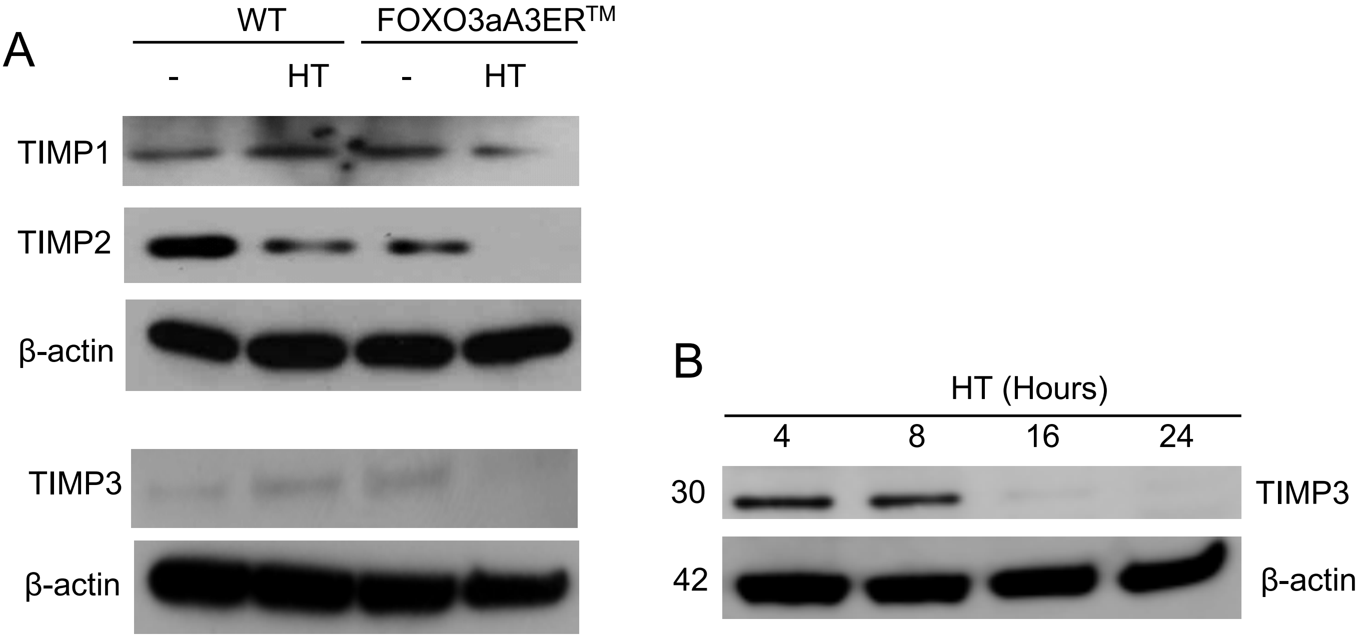

Supplemental Figure II
(A) Western blot of lysates from wild type (WT) control or FOXO3aA3ER^{TM} VSMCs treated with the ethanol carrier control (-) or HT at 24h. **(B))** Western blot of lysates from FOXO3aA3ER^{TM} VSMCs treated with HT from 4-24h. Representative of n=3.

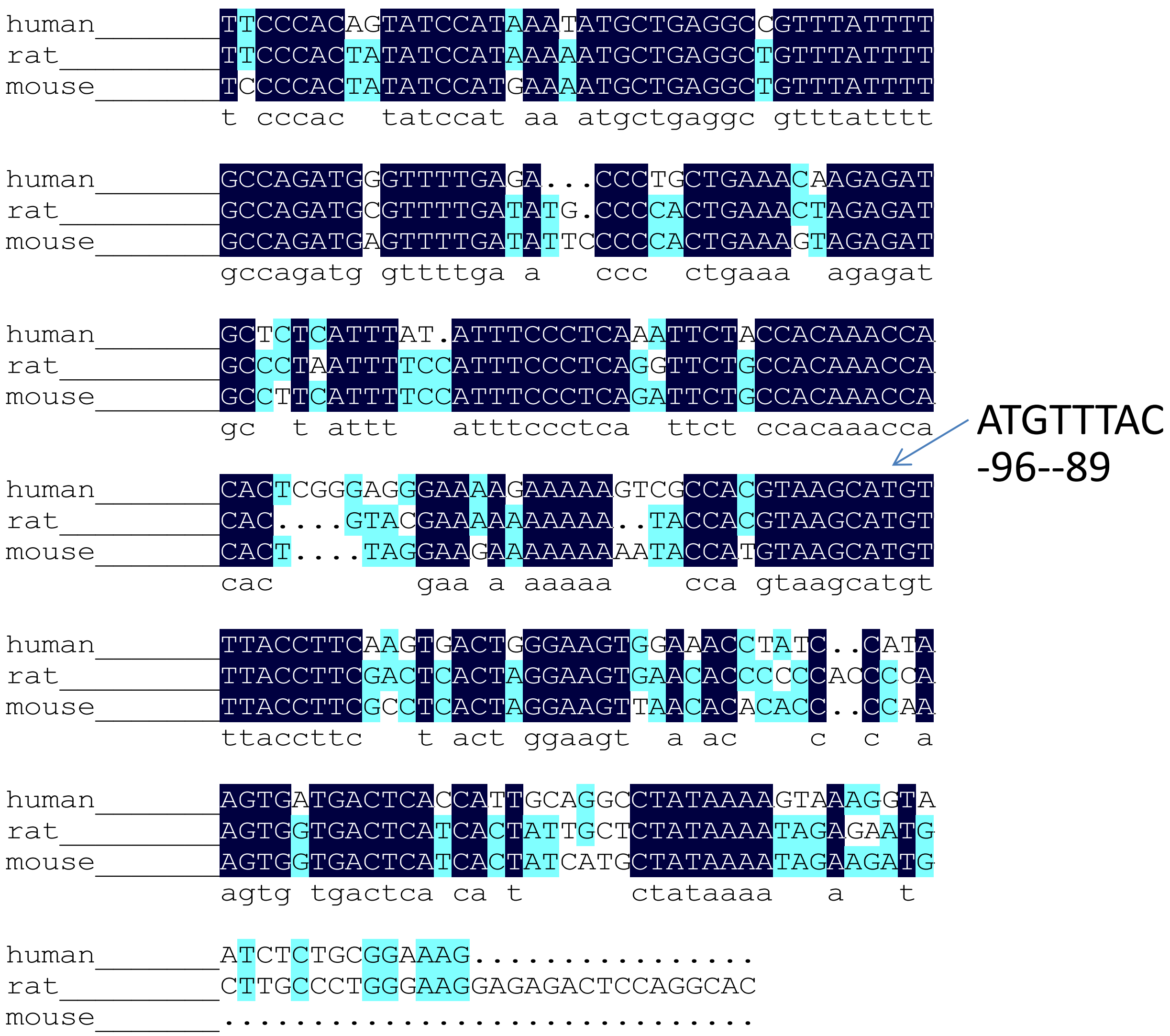

Supplemental Figure III

MMP13 promoter proximal site (-96 bp upstream of start codon) contains a complete FOXO3a DNA binding motif ATGTTTAC. Residues shaded dark blue are conserved between species.

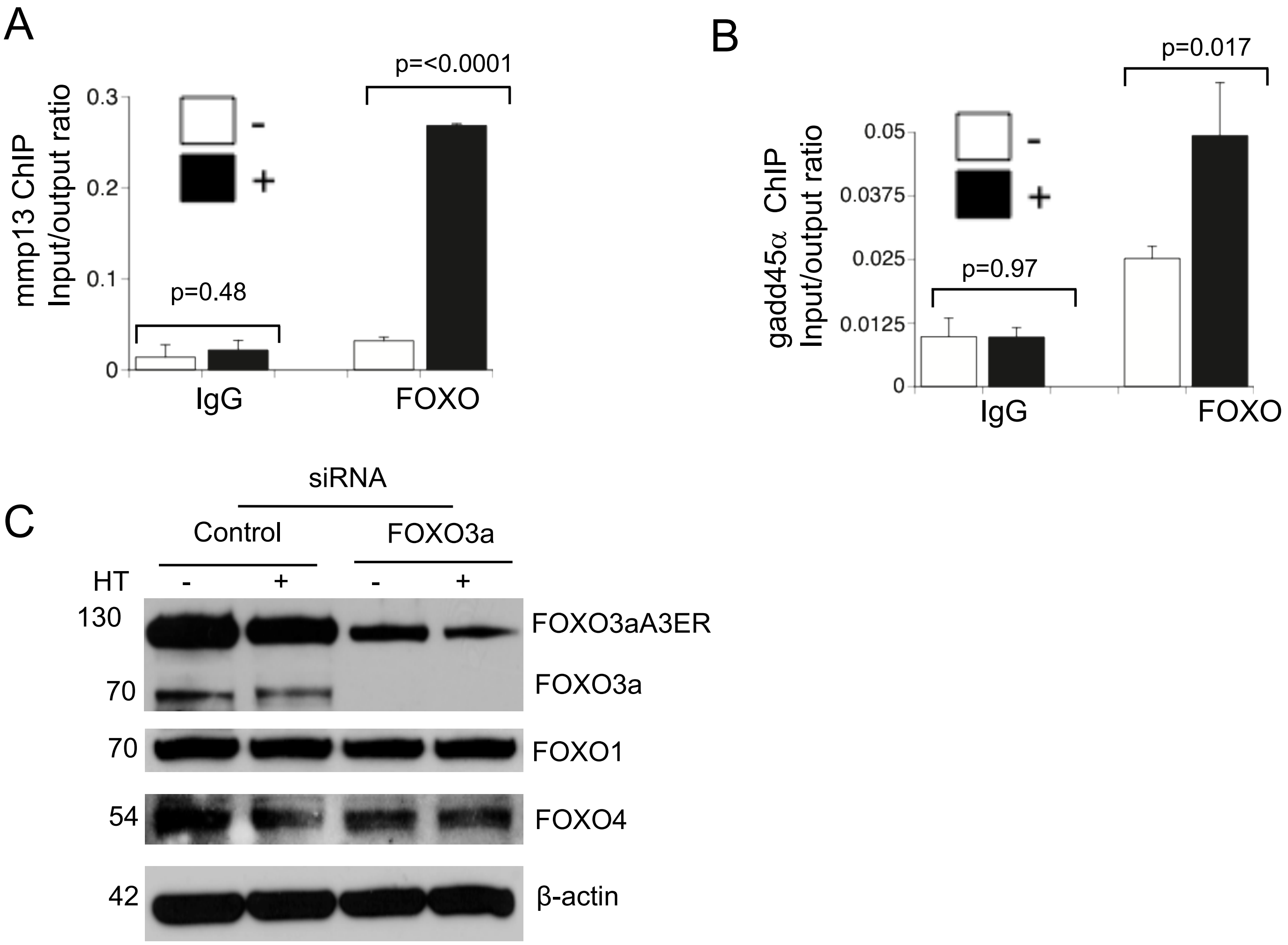

Supplemental Figure IV

(A-B) Chromatin IP (ChIP) for binding of FOXO3a to the mmp13 **(A)** or gadd45α **(B)** promoter in FOXO3aA3ER^{TM} VSMCs treated with carrier control (-) or HT (+) for 24h and immunoprecipitated with an IgG control or MMP13 antibody. **(C)** Western blot for FOXO species in lysates of FOXO3aA3ER^{TM} VSMCs after transfection with either control siRNA (siCtl) or siRNA to human FOXO3a ± 24h treatment with HT.

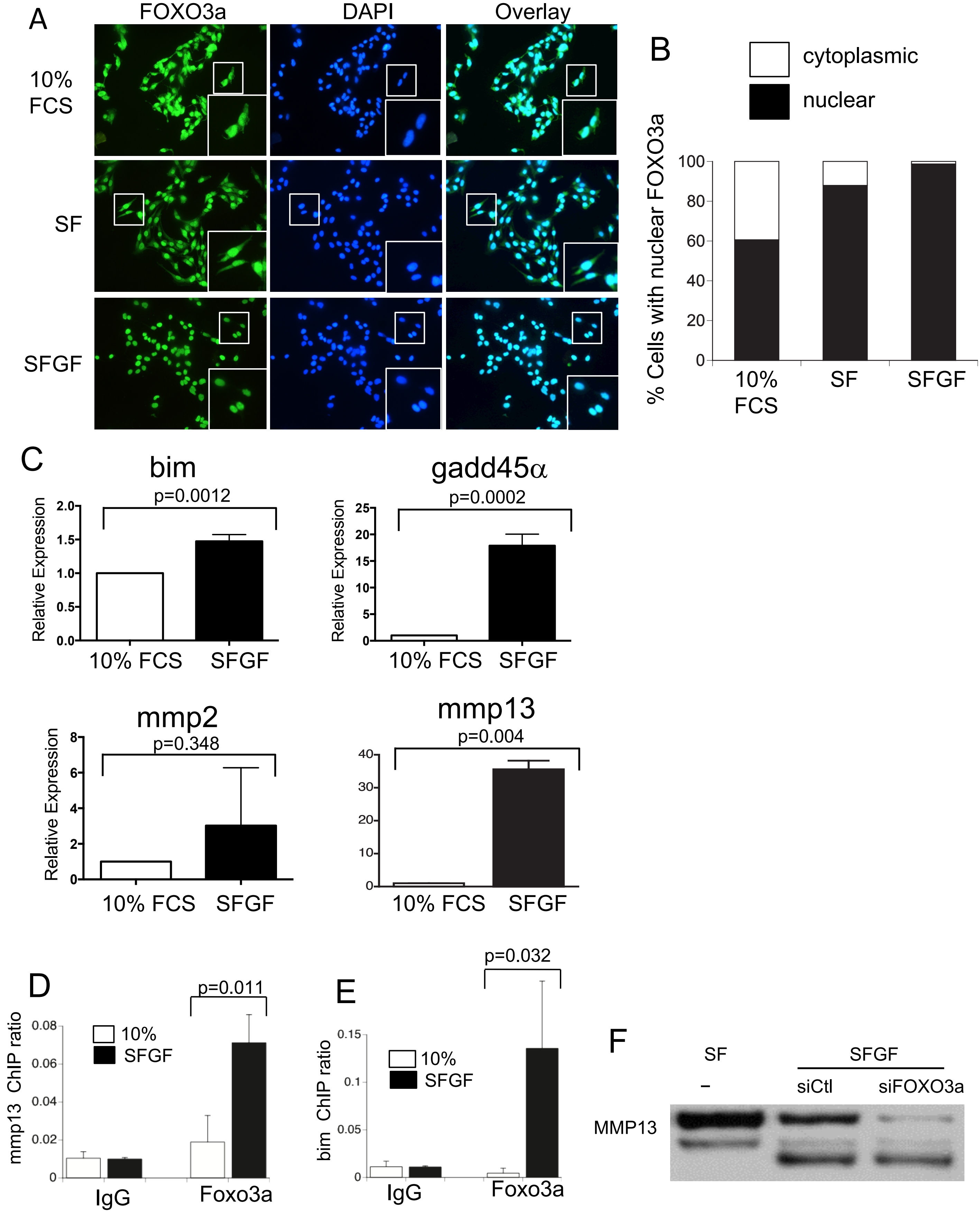

Supplemental Figure V. Nutrient withdrawal induces Foxo3a transcriptional targets (A-B). FOXO3a localization in human VSMCs in 10% FCS, or serum-free (SF) or serum-free/ glucose free media (SFGF) for 24h. Insets show high power view of outlined areas. **(C)** qPCR for bim, gadd45α, mmp2 and mmp13 in control VSMCs in 10% FCS, or 24h after transfer to SFGF media. **(D)** Chromatin IP (ChIP) for FOXO3a binding to the mmp13 **(D)** or bim **(E)** promoter in FOXO3aA3ER^{TM} VSMCs in 10% FCS or SFGF medium, immunoprecipitated with an IgG control or MMP13 antibody. **(F)** Western blot of conditioned medium from human VSMCs transfected with siRNA to control sequences (siCtl) or FOXO3a, transferred to SF or SFGF medium for 24h. Data are means±SD. n=3.

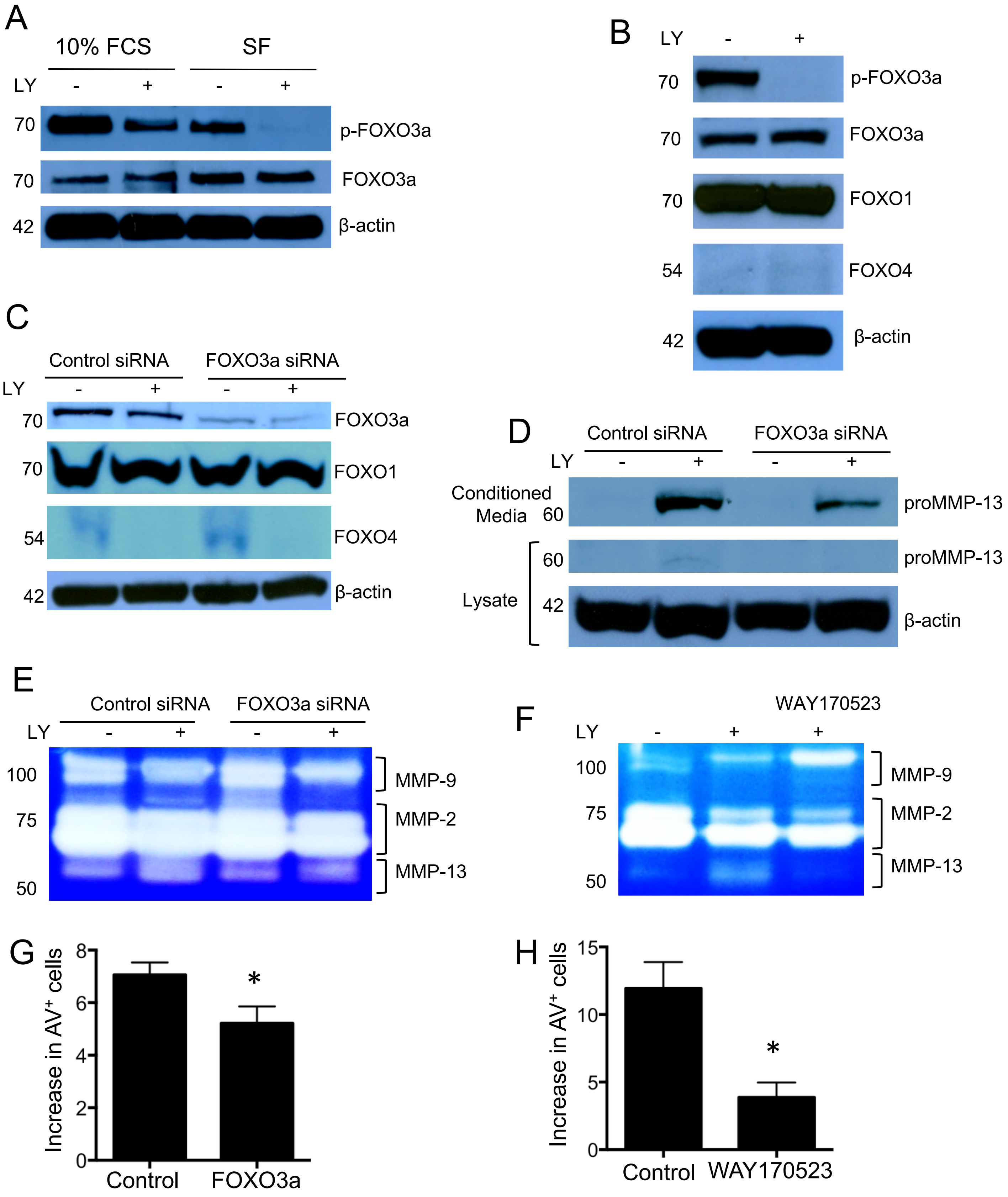

Supplementary Figure VI. PI-3K inhibition activates FOXO3 and induces MMP13 in human VSMCs **(A)** Western blot of cell lysates from human VSMCs treated with 10%FCS or 0%FCS medium (serum-free – SF) ± LY294002 (LY) for 24 hours. **(B-C)** Western blot for FOXO species ± LY294002 (LY) for 24h **(B) or** after prior treatment with control siRNA or siRNA to FOXO3a for 48h ± LY294002 (LY) for 24h **(C)**. **(D-E)** Western blot for MMP13 **(D)** or zymogram **(E)** of conditioned media of human VSMCs ± LY294002 (LY) for 24 hours or additionally after prior treatment with control siRNA or siRNA to FOXO3a. **(F)** Zymogram of human VSMCs ± LY294002 (LY) or after treatment with WAY170523. **(G-H)** Increase in Annexin V-positive cells after treatment of human VSMCs with LY after prior treatment with control siRNA or siRNA to FOXO3a **(G)**, or after treatment with WAY170523 **(H).** Data are means ± SEM. Representative of n=3. *p<0.05 vs. Control.

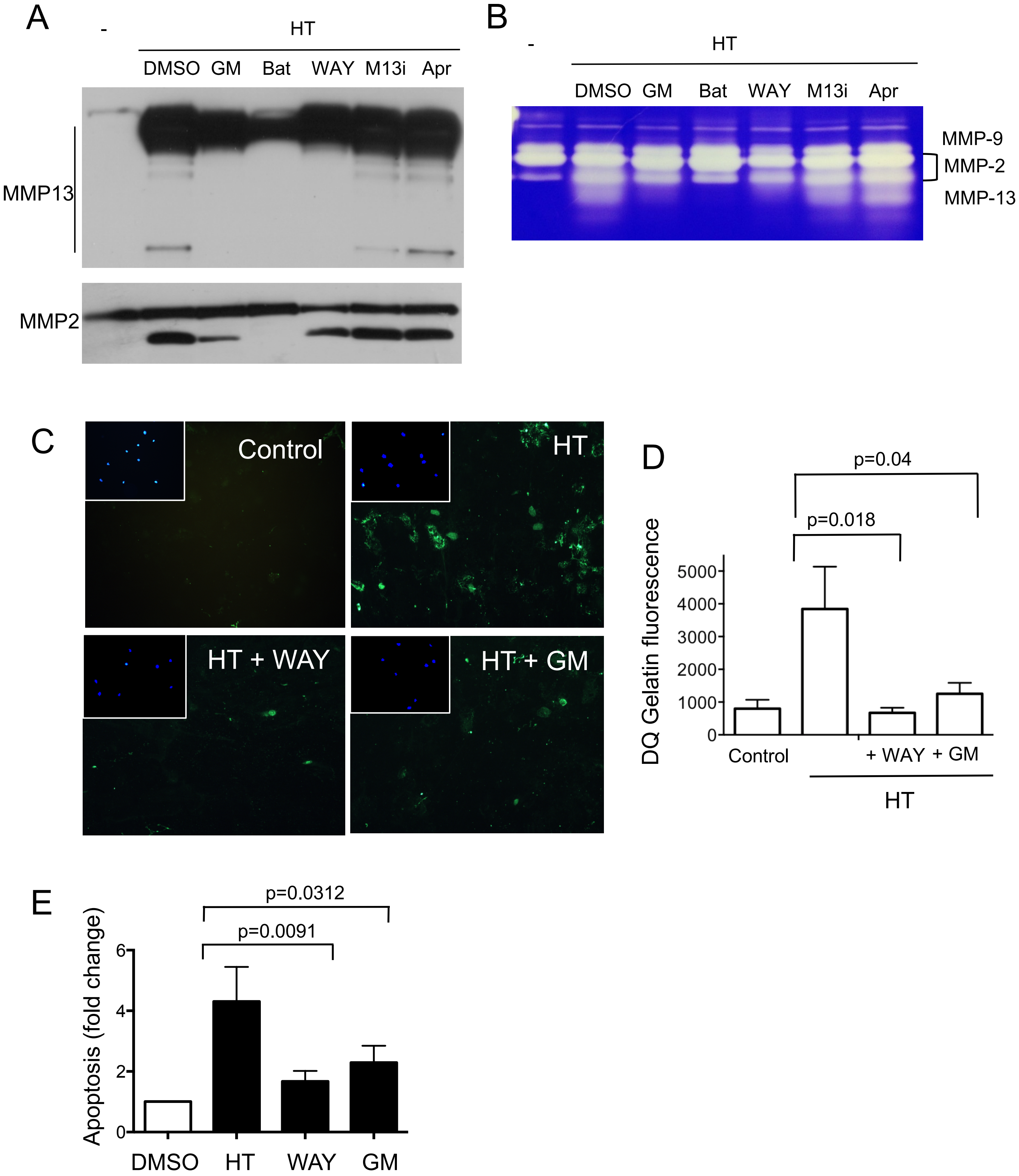

Supplemental Figure VII. MMP13 promotes apoptosis induced by FOXO3a
(A) Western blot for MMP13 or MMP2 in conditioned media of FOXO3aA3ER^{TM} VSMCs after 24h treatment with HT or control (-), with pre-incubation with 10mM GM6001 (GM), 10μM Batamistat (Bat), 10μM WAY170523 (WAY), 1μM M13i or 2mg/ml Aprotinin, or DMSO control. **(B)** Zymogram of conditioned media from VSMCs treated in **(A)**. **(C-D)** DQ-gelatin fluorescence of FOXO3aA3ER^{TM} VSMCs treated with HT ± WAY or GM for 16h. Insets show DAPI of same field as DQ-gelatin. **(E)** Apoptosis of FOXO3aA3ER^{TM} VSMCs after HT ± WAY170523 or GM6001 treatment for 24h. Data are means ± SD.

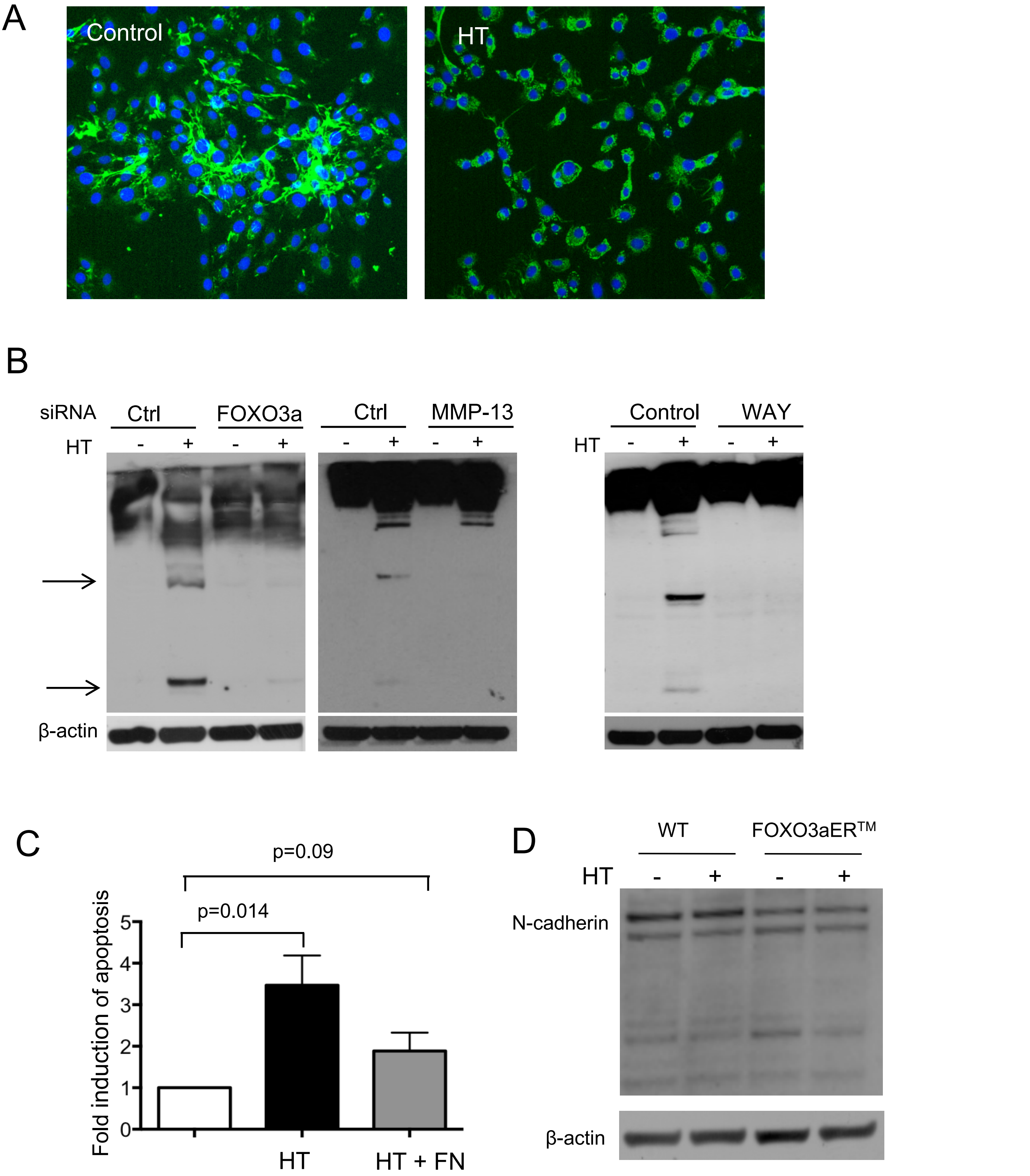

Supplemental Figure VIII. FOXO3a-induced MMP13-mediated cleavage of fibronectin promotes VSMC apoptosis

(A). Immunocytochemistry for fibronectin in cultures of FOXO3aA3ER[TM] cells ± HT for 24h. **(B)** Western blot for fibronectin (arrows) in FOXO3aA3ER[TM] cells ± HT for 24h, preincubated with siRNA to control sequences (Ctrl), FOXO3a or MMP13, or treated with vehicle control or WAY170523. **(C)** Apoptosis of FOXO3aA3ER[TM] VSMCs 24h after HT ± fibronectin (FN) coating of the culture plate. **(D)** Western blot for N-cadherin in wild type control (WT) or FOXO3aA3ER[TM] cells ± HT for 24h. Data are Means ± SD, n=3.

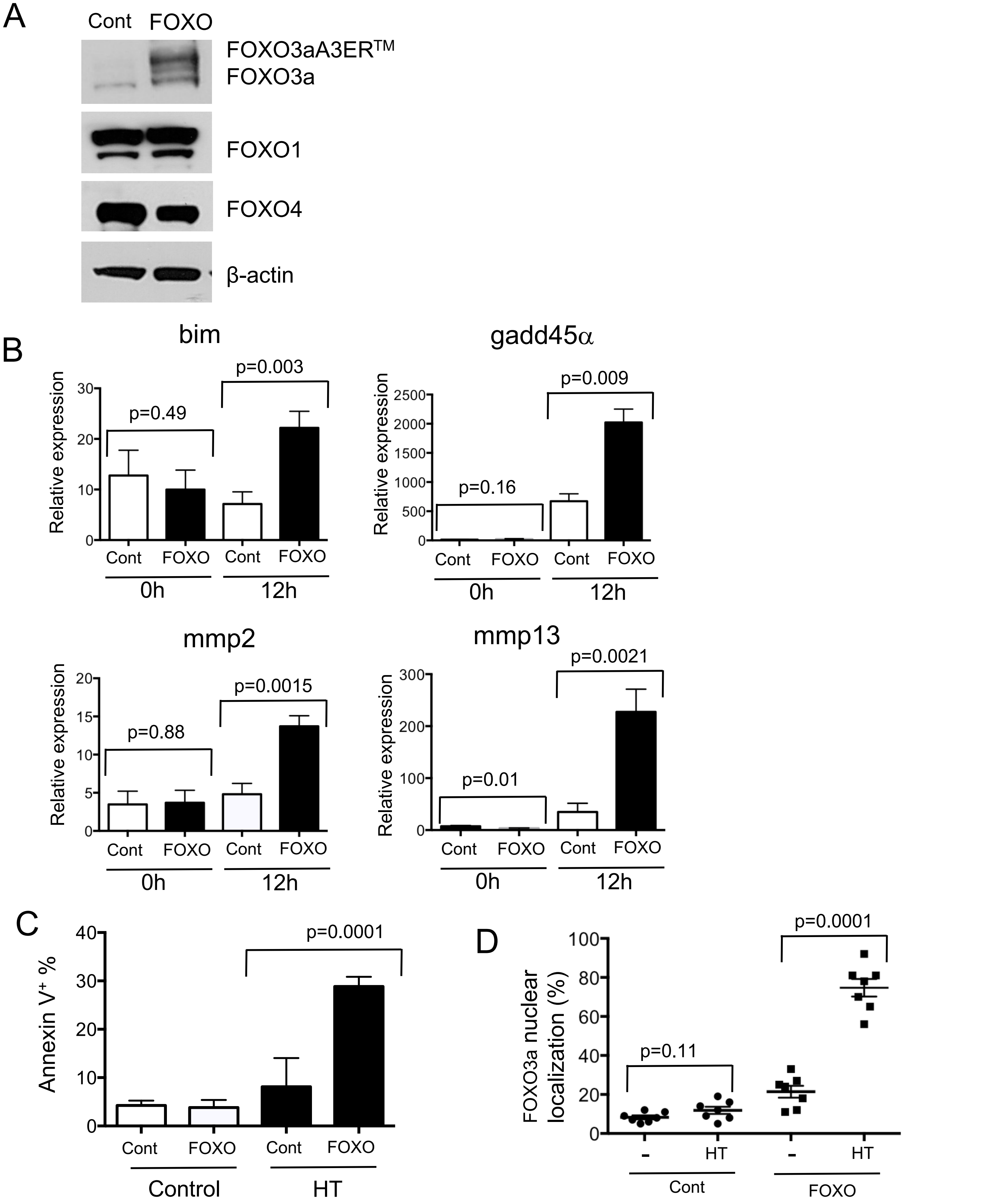

Supplemental Figure IX. SM22aFOXO3aA3ER™ mouse VSMCs induce Foxo3a targets and apoptosis after HT

(A) Western blot for FOXO species in VSMCs derived from littermate control (Cont) or SM22aFOXO3aA3ER™ (FOXO) mice 24h after treatment with HT**.** **(B)** Relative expression by qPCR for bim, gadd45α, mmp2 and mmp13 in VSMCs derived from mice in **(A)** at time 0 or 12hr after 1μM HT. **(C)** Apoptosis (Annexin V$^+$) % determined by flow cytometry of VSMCs derived from mice in **(A)** treated with ethanol carrier (Control) or 1μM HT for 24h. **(D)** Quantification of nuclear FOXO3a in littermate control (Cont) or SM22aFOXO3aA3ER™ (FOXO) mice 24h after treatment with HT. Data are means±SD. N=3 mice in **(A-C)** or n=7 for **(D)**.

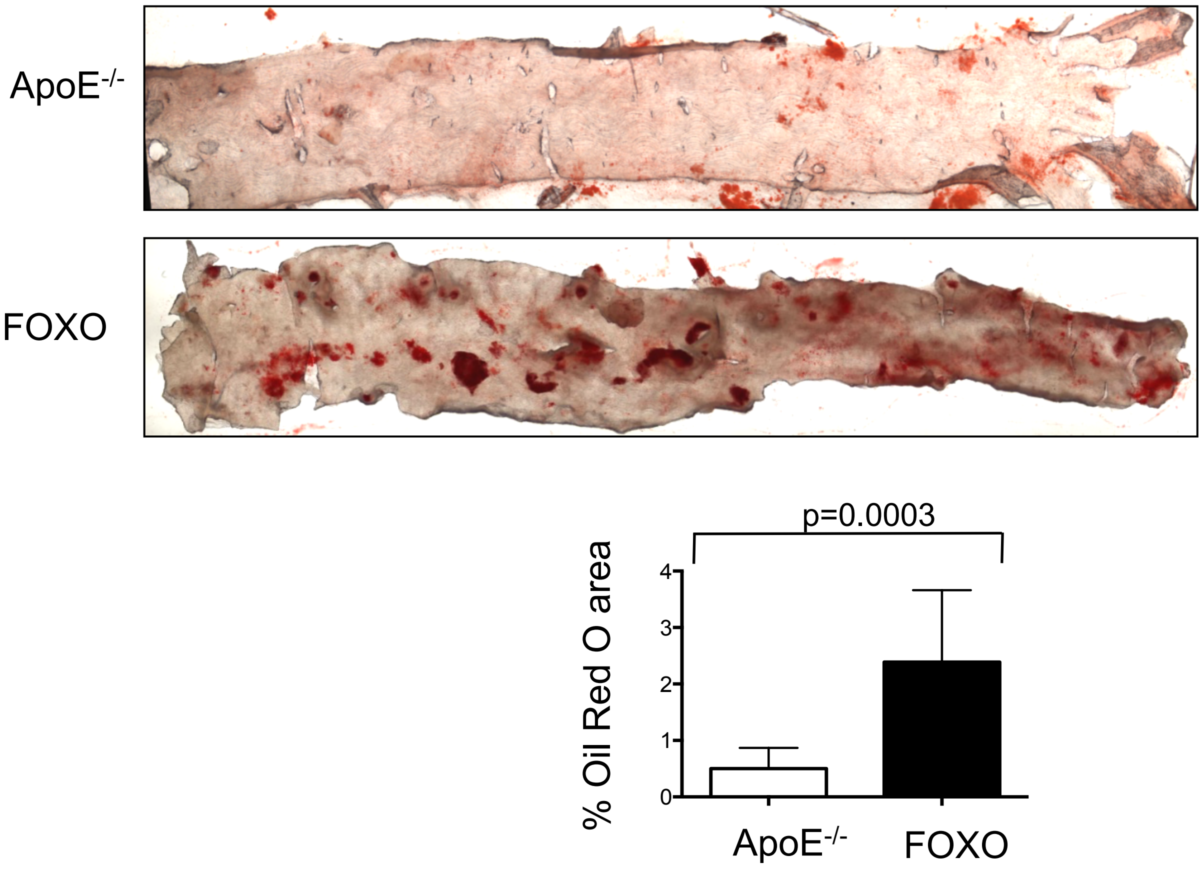

Supplemental Figure X
Oil Red O staining of descending aorta of of Control ApoE$^{-/-}$ (ApoE$^{-/-}$)(n=13) or SM22αFOXO3aA3ER^{TM}/ ApoE$^{-/-}$ (FOXO)(n=14) mice after 14 weeks of fat feeding. Data are means±SD.

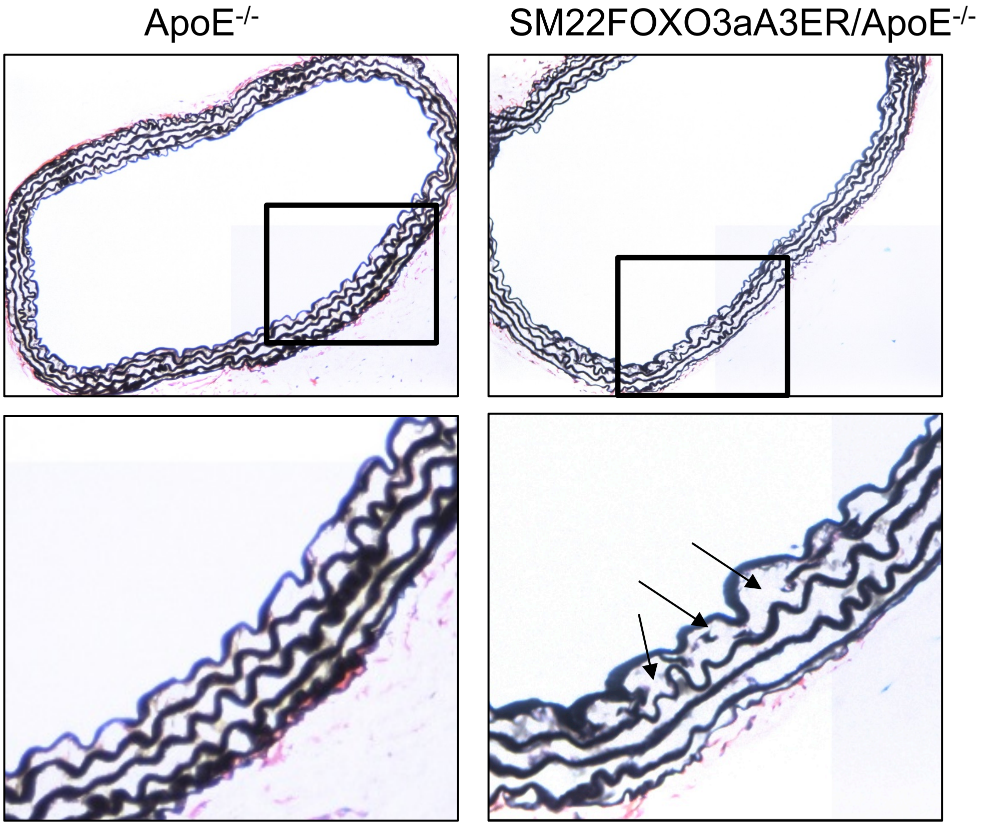

Supplemental Figure XI

Verhoeff Van Gieson stain for elastin in littermate control and SM22αFOXO3aA3ER^{TM}/ApoE$^{-/-}$ mice, showing elastin breaks (arrows).

Up-regulated genes

Gene name	Encoded protein	Fold change	Potential function
MMP13	Matrix metallopeptidase 13	371.37	ECM degradation
BMP2	Bone morphogenetic protein 2	9.80	MMP activator
Cited2	Cbp/p300-interacting transactivator 2	8.96	apoptosis
CXCL10	Interferon gamma-induced protein 10	7.88	Chemoattractant
Bcl2l11	Bcl-2-like protein 11, BIM	6.24	apoptosis
Bcl2	B-cell lymphoma 2	6.24	apoptosis
Adamts5	A disintegrin and metalloproteinase with thrombospondin motifs, 5	5.93	major aggrecanase in mouse cartilage
Hmga2	The high mobility group A 2	5.39	necrosis
Gadd45	Growth Arrest and DNA Damage	4.29	DNA repair
Pim1	Proto-oncogene protein kinase	3.67	apoptosis
Bcl6	B-cell lymphoma 6 protein	3.41	apoptosis
CDKNIB	p27	2.85	growth arrest
Adam17	ADAM metallopeptidase domain 17	2.00	Sheddase
Pink1	PTEN-induced putative kinase 1	2.00	apoptosis

Down-regulated genes

Gene name	Encoded protein	Fold change	Potential function
TIMP3	tissue inhibitor of metalloproteinase 3	-27.16	MMP inhibitor
WISP2	WNT1-inducible-signaling pathway protein 2	-7.10	Akt activator and apoptosis inhibition
Itga11	Integrin alpha-11	-5.54	Cell attachment to ECM
Cdh13	T-cadherin	-5.18	Cell adhesion
Tagln	SM22α	-4.00	SMC contractile marker
Cnn1	Calponin 1	-4.02	SMC contractile marker
WISP1	WNT1-inducible-signaling pathway protein 1	-3.42	Akt activator and apoptosis inhibition
Itga7	Integrin alpha-7	-2.96	Cell attachment to ECM
Ccnd1	G1/S-specific cyclin-D1	-2.72	Cell cycle progression
smtn	Smoothelin	-2.12	SMC contractile marker
Itga5	Integrin alpha-5	-2.00	Cell attachment to ECM
Myh10	SMC myosin heavy chain isoform	-2.00	SMC contractile marker
des	Desmin	-2.00	SMC contractile marker

Supplemental Table I.
Most upregulated and down-regulated genes in FOXO3aA3ER^{TM} vs. control VSMCs 4 hours after HT.

Name	Promoter sequence	Comments
pMMP13-luc	-1600 - +1	
pMMP13M-luc	-1600 - +1 mutant	Site directed mutagenesis of above
pMMP2-luc	-1545 - +1	
pMMP3-luc	-1551 - +39	
pMMP9-luc	-1775 - +1	
pFHRE-luc	-743 - -648	
pRL-CMV	7-803	

Supplemental Table II. MMP or Foxo response element reporter plasmids
Sequence numbers are relative to the transcriptional start site.

	$ApoE^{-/-}$	FOXO	p value
**Cholesterol (mmol/L)**			
0	7.68±1.99	6.94±1.16	0.25
7	10.77±1.86	10.68±1.25	0.73
14	13.30±2.33	13.33±1.78	0.81
**Triglyceride (mmol/L)**			
0	1.17±0.26	1.22±0.36	0.62
7	0.73±0.15	0.78±0.14	0.40
14	1.28±0.36	1.13±0.28	0.24
**LDL (mmol/L)**			
0	6.60±1.91	5.78±1.11	0.19
7	10.16±1.85	10.02±1.18	0.65
14	12.38±2.15	12.54±1.66	0.92
**HDL (mmol/L)**			
0	0.55±0.20	0.61±0.17	0.48
7	0.28±0.07	0.31±0.04	0.099
14	0.34±0.10	0.29±0.05	0.10

Supplemental Table III

Serum lipids in $ApoE^{-/-}$ littermate control ($ApoE^{-/-}$)(n=13) or SM22αFOXO3aA3ER^{TM}/$ApoE^{-/-}$ (FOXO)(n=14) mice at 0, 7 and 14w of high fat diet and HT administration. Data are means±SD.

	Systolic (mmHg)	Diastolic (mmHg)	Mean (mmHg)	HR (beats/min)
**$ApoE^{-/-}$**	107.51±11	58.32±13	68.78±13	659.77±33
**FOXO**	113.89±10	56.32±12	68.44±12	626.44±48
**p value**	0.151	0.694	0.946	0.056

Supplemental Table IV

Blood pressure and heart rate of $ApoE^{-/-}$ littermate control ($ApoE^{-/-}$)(n=13) or SM22αFOXO3aA3ER^{TM}/$ApoE^{-/-}$ (FOXO) mice (n=14) after 14 weeks high fat diet and HT administration. Data are means±SD.

Cytokine (pg/mL)	$ApoE^{-/-}$	FOXO	p value
CXCL-9	80.60±39.97	109.37±86.39	0.47
IFN-γ	1.00±0.42	1.43±1.82	0.57
IL-10	47.49±17.15	43.91±6.53	0.62
IL-1β	1.26±0.59	1.03±0.32	0.37
IL-2	2.29±1.01	2.35±1.09	0.88
IL-5	25.04±9.57	23.68±8.83	0.79
IL-6	73.42±45.54	69.96±40.71	0.89
TNF-α	22.47±8.31	22.26±4.53	0.96

Supplemental Table V

Serum cytokines in $ApoE^{-/-}$ littermate control ($ApoE^{-/-}$)(n=6) or SM22αFOXO3aA3ER^{TM}/$ApoE^{-/-}$ (FOXO) mice (n=7) after 14 weeks high fat diet and HT administration. Data are means±SD.

	Control ($ApoE^{-/-}$)	SM22α-FOXO3aER^{TM}/$ApoE^{-/-}$	p Value
**Aortic root**			
Plaque area (μm^2)	307808 ± 33937	490227 ± 41346	**0.0024**
Necrotic core area (μm^2)	67723 ± 12840	203535 ± 36488	**0.0022**
Core/Plaque ratio	0.219±0.02	0.402±0.06	**0.014**
Cap area (μm^2)	72111 ± 11377	50207 ± 10319	0.1653
Cap cell count	181.3 ± 26.94	152.1 ± 39.66	0.5536
Cap/Core ratio	1.479 ± 0.40	0.265 ± 0.026	**0.0044**
Cap/Plaque ratio	0.225±0.02	0.0986±0.02	**0.0001**
TUNEL (% cells)	6.154 ± 1.480	19.08 ± 1.943	**< 0.0001**
BrdU (% cells)	3.45 ± 1.58	4.24 ±2.61	0.191

Supplemental Table VI
Morphometric and immunohistochemical quantification of aortic root atherosclerotic plaques in Control $ApoE^{-/-}$ and SM22a-FOXO3aER$^{TM/}$$ApoE^{-/-}$ mice at 22w after fat feeding for 14w and Tamoxifen administration. Data are means±SD (n=11-14).

	Control + vehicle	Control + WAY	p value vs. vehicle control	SM22α-FOXO3aER^{TM} + vehicle	SM22α-FOXO3aER^{TM} + WAY	P value vs. vehicle control
Lumen area (μm^2)	34710±7543	22440±2856	0.079	24383±6310	34943±11564	0.223
Intimal area (μm^2)	17222±4726	8376±3731	0.083	37301±9442	9886±4552	**0.011**
Medial area (μm^2)	40291±6299	49636±4850	0.130	40926±4437	62958±16227	0.120
Intimal Cell count	127±31	106±39	0.346	331±90	130±59	**0.043**
Medial Cell count	164±23	147±12	0.260	196±26	153±27	0.138
Intimal Cellularity ($cells/mm^2$)	11765±1807	14681±1700	0.131	10511±703	13006±423	**0.005**
Medial Cellularity ($cells/mm^2$)	4617±613	3016±197	0.018	4818±325	2833±418	**0.003**
Cell proliferation (No of cells)	197.7±63.9	166.8±81.3	0.38	611.4±144.1	115.8±89.0	**0.027**
Apoptosis (No of cells)	1.1±0.5	0.0±0.0	**0.020**	2.4±0.5	0.6±0.6	**0.024**

Supplemental Table VII
Morphometry of left carotid artery at 28 days post ligation of FOXO3aA3ER^{TM} or littermate control mice, treated with HT throughout ± WAY170523 or vehicle control from 0-7 days. Cell proliferation and apoptosis represent number of cells positive for BrdU or TUNEL in both intima and media. Data are means±SEM, n=6-11.
