# Supplementary figures and images for: FOXO3a (Forkhead Transcription Factor O Subfamily Member 3a) Links Vascular Smooth Muscle Cell Apoptosis, Matrix Breakdown, Atherosclerosis, and Vascular Remodeling Through a Novel Pathway Involving MMP13 (Matrix Metalloproteinase 13)
Source: Arterioscler Thromb Vasc Biol. 2018 Jan 11;38(3):555–65. doi: 10.1161/ATVBAHA.117.310502 (PMC5828387; doi:10.1161/ATVBAHA.117.310502)

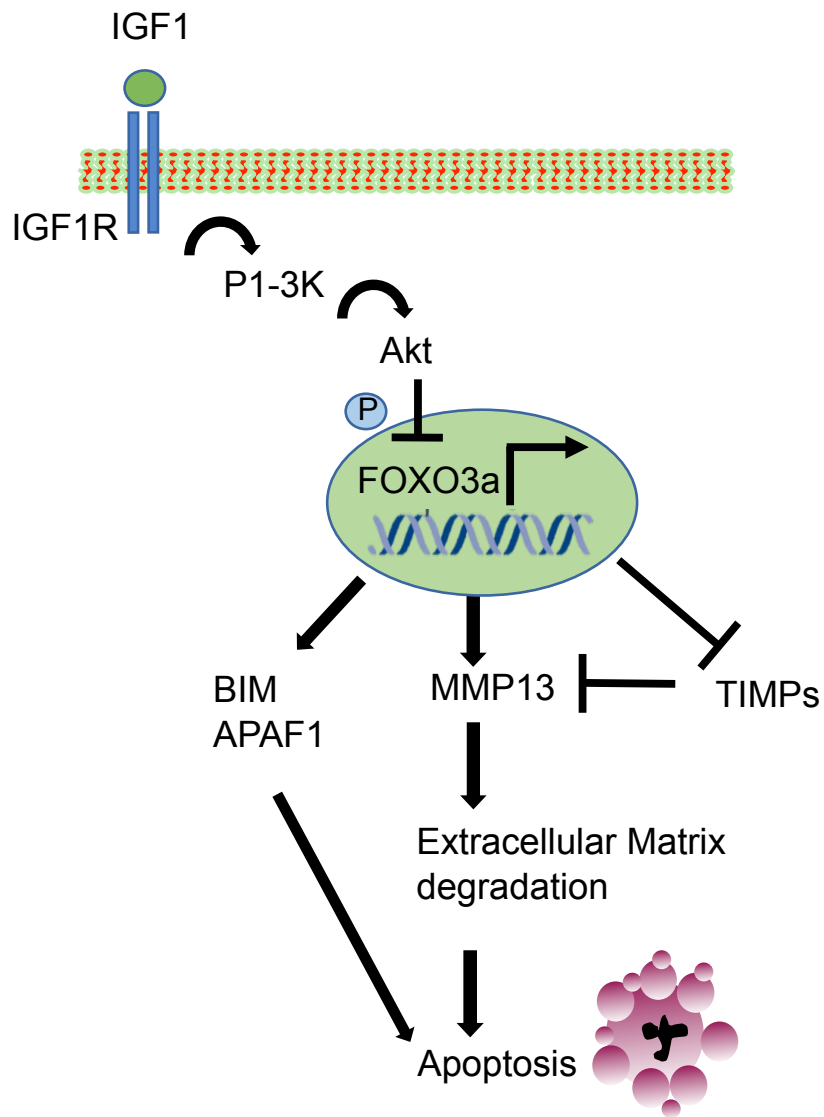

Supplement: Supplementary file 3 [file atv-38-555-s003.pdf]
